# A systematic and critical review of measurement scales for evaluating gastrointestinal symptoms during exercise

**DOI:** 10.1080/15502783.2026.2735563

**Published:** 2026-09-29

**Authors:** Robyn Aitkenhead, Mark Waldron, Gillian E. Conway, Katy Horner, Shane M. Heffernan

**Affiliations:** a Applied Sports Science Technology and Medicine Research Centre (A-STEM), Faculty of Science and Engineering, Swansea University, Swansea, UK; b Welsh Institute of Performance Science, Swansea University, Swansea, UK; c School of Health and Behavioural Sciences, University of the Sunshine Coast, Queensland, Australia; d In Vitro Toxicology Group, Institute of Life Science, Swansea University Medical School, Swansea, UK; e School of Public Health, Physiotherapy and Sport Science and UCD Institute of Food and Health, University College Dublin, Dublin, Ireland

**Keywords:** Gastrointestinal symptoms, Likert scales, exercise tolerance, validation

## Abstract

**Background:**

Exercise-induced gastrointestinal symptoms (GIS) are commonly reported, with either Likert-type scales or visual analog scales (VAS) used to quantify the incidence and magnitude of symptoms. However, these measurement tools lack consistency in their design, reporting, and key aspects such as the validity of these tools is unclear. This study aimed to systematically review and critically discuss the various Likert-type and VAS scales used to assess GIS during exercise.

**Methods:**

A search conducted in August 2025, in accordance with PRISMA guidelines, identified full-text articles evaluating GIS during exercise or sport.

**Results:**

In total, 126 studies were included, representing 50 different scales and 13 scale lengths. The most frequently used measures were 11-point Likert-type scales and VAS (46%). Only one study assessed the reliability of an 11-point exercise-specific scale, and only one study used a validated retrospective questionnaire developed for assessing chronic GIS in runners. Most studies (92%) did not justify their scale selection, often adapting tools from non-exercise contexts or using custom-designed measures.

**Conclusion:**

The current review highlights substantial heterogeneity in GIS measurement during exercise and a paucity of evidence on validity and reliability. In addition to identifying current methodological limitations, the current review provides practical recommendations to support improved GIS assessment and reporting in exercise settings. The development of a standardized, fully validated exercise-specific GIS scale remains a priority, as current tools range from those with limited (partial) psychometric evaluation to those with no formal validation evidence, thereby limiting consistency, reliability, and comparability across studies and in practice.

## 1. Introduction

In the last two decades, there has been a significant growth in the investigation of gastrointestinal symptoms (GIS) during exercise (Figure 1). These symptoms, ranging from mild to severe, have attracted significant attention and have ostensibly provided some understanding of the prevalence, severity, and impact of GIS during exercise [1–3]. Symptoms can occur in both the upper- (esophagus and stomach) and the lower-GI tract (small bowel and colon) [4], and the severity of the GI disturbance can have an adverse effect on exercise performance, potentially leading to early withdrawal from training activities or competition [5]. Most studies use Likert scales and/or visual analog scales (VAS) to measure individuals' perception of GIS, measured across a wide range of exercise modalities to mitigate, manage, and/or treat GIS [6,7]. In addition, GIS measurement occurs in both laboratory settings and in real-world environments, such as marathons and triathlons [8–10]. Therefore, it is reasonable to consider that exercise mode and the environment of data collection may be a factor for scale selection.

Likert-type scales are widely utilized tools to measure feelings, perceptions, and attitudes [11]. Likert scales typically present respondents with a series of statements or items accompanied by worded anchors, allowing them to indicate their level of agreement or disagreement [11,12]. In contrast, VAS provide a continuous rating scale, represented by a straight line [12]. Instead of discrete response options, respondents mark a point along the line to indicate the intensity or magnitude of an experience or symptom, such as pain [13]. General gastroenterology research utilizes many validated Likert-type scales [14,15] for identifying chronic GIS that have been validated on 10-point [14], 7-point [15] and 5-point [16] scales, for specific and distinct purposes. The 100-mm VAS [17] has been referred to as the “gold standard” tool for clinical gastroenterology cases [18,19]. However, most scales appear to be unreferenced and/or are adapted from previous studies and the absence of a fully validated scale for measuring GIS during exercise is a significant concern.

As a result of the wide variation in scales applied within the literature (Figure 1), there are additional concerns regarding the use of GIS scales, particularly the appropriateness of directly comparing results obtained from GIS scales of varying lengths, types, and taxonomies. These scales were not designed to accommodate such levels of flexibility or interoperability. Additionally, differences in magnitude scaling, descriptors, and taxonomy between these scales can lead to discrepancies in interpreting severity levels. For instance, some studies have classified symptoms as “severe” when the severity score was >4 [4,20] and >5 [10,19,21] on the same 10 point scale, thus complicating direct comparisons between studies. These discrepancies raise concerns about whether different scales can be used interchangeably. It is feasible that seemingly “small” errors in severity classification could significantly affect clinical decisions, potentially altering an individual's treatment or intervention pathway. These inconsistencies become clinically consequential when considering the intervention thresholds proposed by [22], where a single point discrepancy (e.g. 4 vs. 5 on a 10-point scale) could determine whether an athlete receives basic hydration advice or targeted gut-training protocols, potentially altering symptom progression and exercise tolerance. Thus, highlighting the importance of standardizing the use of validated scales to ensure consistency and accurate interpretation of exercise-induced GIS severity across studies and clinical contexts.

The severity and occurrence of GIS during exercise can vary significantly between individuals and is an important factor to consider when selecting a scale. Measurement of GIS is subjective, and, therefore, the participants' proper interpretation of the descriptors is necessary to ensure that the GIS measurements are reported reliably and validly. That is; respondents must possess a uniform understanding of the construct in question and how this is to be perceived. This may be challenging, given the many different descriptors currently used to represent GIS magnitude. This includes descriptors such as “hardly any complaint”, “not at all” or “very comfortable” observed at points 0 or 1 on a scale [23–25]. The underlying construct being examined is uncertain when use of descriptors such as “comfort” and “complaint” are associated with a similar GIS magnitude, and interchangeable use of such scales assumes that the removal of a complaint is equivalent to achieving high levels of comfort, which is also questionable. Indeed, the extreme end of GIS scales encompass a much wider range of terms, such as “severe complaint”, “very, very much”, “worst it has ever been” or “unbearable” [4,23,26]. Variability in descriptors (e.g. “comfort” vs. “complaint”) across GIS scales introduces uncertainty about the construct being measured. This risks conflating distinct experiences (e.g. functional impairment vs. subjective discomfort) and undermines the validity of cross-study comparisons [27]. Addressing these inconsistencies remain a key challenge for advancing research on exercise-induced GIS measurement.

Despite recent calls to standardize GIS assessment during exercise [18], current practices remain inconsistent and lack foundational validation. Unlike clinical scales that adhere to rigorous development frameworks [28], existing exercise-GIS scales appear to frequently omit critical validation steps: (1) content validity testing via structured expert and athlete evaluations to ensure symptom relevance and comprehensiveness; (2) construct validity confirmation through factor analysis, and (3) reliability assessments beyond basic test-retest consistency, such as internal consistency or interrater reliability [29–31]. Without these, symptom classification remains arbitrary.

This review builds upon existing literature by evaluating the historical progression of GIS measurement tools, and critically discussing current methods and their potential to affect GIS reporting. Specifically, this review aims to systematically and critically assess: (1) the use of measurement tools used to assess GIS during exercise, focusing on the number and distribution of scale points and their impact on symptom severity classification; (2) scale taxonomy to identify inconsistencies in how GIS scales categorize symptoms; (3) symptom descriptors to highlight variability in terminology, definitions, and severity thresholds used across studies; and (4) the statistical methods and reporting practices applied. By addressing these areas through a comprehensive analysis of GIS measurement scales, this review seeks to provide actionable recommendations for developing more robust and standardized tools to advance GIS measurement during exercise.

## 2. Methods

### 2.1. Design and search strategy

The search strategy adhered to the Preferred Reporting Items for Systematic reviews and Meta-Analyzes (PRISMA) guidelines [32]. While the current review focused on GIS measurement scales, it was not designed as a formal systematic review of measurement properties; rather, it aimed to identify, characterize, and critically evaluate the scales used within exercise research. Inclusion criteria were studies in the English language, including healthy adults aged ≥ 18 years, featuring participants who had participated in an exercise trial, intervention, or habitual exercise, with a measurement of a GIS before, during, or after exercise/sport. Exclusion criteria were animal and in-vitro studies, inability to access full-text article, gray literature, studies in humans aged < 18 years or suffering from any disease. All research, regardless of the year of publication, was included, and medical subject heading (MeSH) terms were active. Studies were included regardless of participant training status and sex. The current review was not prospectively registered.

### 2.2. Data selection and collection

Initial screening of titles and abstracts determined suitability for inclusion, the entire article was retrieved for full-text review to determine inclusion. Four electronic databases were searched, including PubMed, Scopus, Science Direct and Sports Discus. A search was carried out in August 2025. The following search terms were used (exercise OR endurance OR “physical activity” OR “exercise training” OR exercise-induced OR “physical function” OR “physical performance” OR “running” OR “cycling”) AND (gastrointestinal OR gut permeability OR gut barrier OR intestine* OR gut dysfunction OR leaky gut OR gut injury OR gut health OR GI symptoms OR GI stress).

### 2.3. Study selection

Retrieved articles were exported to Rayyan [33] for the screening process, where all duplicates were removed. Titles and abstracts were screened by two reviewers (RA, SH), and the remaining articles were reviewed for full-text screening by two reviewers (RA, SH). Any inclusion disagreements were discussed between the two reviewers, resulting in 100% agreement for included articles. The reasons for removal are detailed in a PRISMA flow diagram (Figure 1).

### 2.4. Data extraction

Data were extracted into a standardized table (Table 1). The extracted data were appropriate for review, including participant characteristics, exercise intervention, the GI scale used, frequency of recording, rationale for scale selection, the symptoms recorded and the statistical methods (Table 1). Exercise data were extracted as mode, intensity (expressed as percentage of oxygen uptake, maximal power, time trial, or time to exhaustion). Regarding GI scales, extraction included the measurement tool used, the length, anchors used for severity rating and the terminology. In this review, VAS were classified based on their presentation as a continuous line marked in centimetres (cm) or millimeters (mm). Participants of these studies were instructed to indicate their response by marking a point along this line to represent the magnitude of the symptom being assessed. Likert scales, on the other hand, were characterized by the presentation of discrete numerical values at each point on the scale. Participants were typically provided with a set of response options, each associated with a specific number, and were instructed to select the corresponding number that best represented the magnitude of the symptom being assessed. Unlike VAS, Likert scales did not involve marking a point along a continuous line, but rather, participants directly provided a numerical response from the pre-determined values. The scales used in this study were classified into distinct groups based on key taxonomic differences, including categories, descriptors, anchors, and magnitude or length.

**Table 1.** Summary of studies which measured GIS using Likert scales of visual analog scales in an exercise setting (*n* = 126).

Study	Participant characteristics	Exercise intervention	GI scale	Frequency of recording	Rationale for GI scale	Symptoms included	Statistical method selected
Aragón-Vargas et al. [34]	16 participants (7 F, 9 M, 24.3 ± 4.6 yrs)	Cycled at 32 °C and 70% RH for 90 min at 60–75% HR_max_	0 (no problem) to 9 (the worst it has ever been)	Pre/post	Reference: Pfeiffer et al., [4] *Reason for selection:* No	Reflux/heartburn, belching, bloating, stomach cramps/pain, nausea, vomiting, intestinal/lower abdominal cramps, flatulence, urge to defecate, side ache/stitch, loose stool, diarrhea, intestinal bleeding, dizziness, headache, muscle cramp, urge to urinate	*Statistical method*: two-way, repeated-measures ANOVA and logistic regression on each symptom *Data reported*: Difference between conditions
Barber et al. [35]	7 trained male runners (22 (18–30) yrs)	2 h run (60% $\dot{V}$ O_2peak_)	7-point scale, adapted from GSRS	30-min	Reference: Revicki et al., [36] *Reason for selection:* “The GSRS has adequate internal consistence (*α* > 0.61), construct and discriminant validity, and is suitable for comparisons over 6 weeks”	Four questions related to upper, three to central, and two to lower gastrointestinal symptoms	*Statistical method:* repeated measures ANOVA or mixed-effects model as appropriate *Data reported:* Median and 95% CI
Baur et al. [37]	10 trained male cyclists and triathletes (26 ± 8 yrs)	~3 h cycle (1 h at 50% W_max_, 8 × 2-min intervals at 80% W_max_, and 10 maximal sprints)	100-mm Likert scale, nothing at all, extremely weak, very weak, weak or mild, moderate, strong, very strong, extremely strong, and absolute maximum	15-min	Reference: Rowlands et al. [38] and Oosthuyse et al. [38] *Reason for selection:* No	Nausea, abdominal cramp, and fullness	*Statistical method:* Probabilistic magnitude-based inferences *Data reported:* Mean
Bennett et al. [39]	14 trained participants (25 ± 7 yrs)	back-to-back 90-min intermittent high-intensity treadmill running protocols separated by 3 h	5-point scale with word anchors. 0 = no symptoms, 1 = mild, 2 = moderate, 3 = moderately severe, and 4 = severe symptoms	30-min	Reference: No *Reason for selection:* No	Not stated	*Statistical method:* Did not state *Data reported:* mean ± SD
Biondich & Joslin [40]	52 runners (48.9 yrs)	210-km multiday foot race in Sri Lanka	0–5 (none to unbearable)	Pre/post	Reference: Bovenschen et al. [41], Revicki et al. [36] *Reason for selection:* adapted from previously published tools used to measure symptom severity of gastroparesis in the context of chronic illness	Nausea, vomiting, loss of appetite, loss of desire to drink, abdominal “sloshing,” and abdominal pain	*Statistical method:* Did not state *Data reported:* median & range & incidence of GIS (%)
Bordoli et al. 42	16 endurance-trained male cyclists (26 ± 6 yrs)	Exercise performance test, comprising 5 repeated blocks consisting of 1 and 4 km TT interspersed with 10 min of moderate-intensity exercise	0 to 10, with 0 being “no symptom” and 10 being “severe symptom”	Pre/during/post	Reference: Carr et al. [43], Miller et al. [44] *Reason for selection: No*	Nausea, flatulence, stomach cramping, belching, stomach-ache, bowel urgency, diarrhea, vomiting, and stomach bloating	*Statistical method*: Pairwise comparisons *Data reported*: Not reported
Burdon et al. [45]	10 trained males (30 ± 7 yrs)	90-min of steady state cycling (SS; 62% $\dot{V}$ O_2max_) in the heat (32.1 ± 0.9 °C, 40 ± 2.4% RH)	10 cm VAS	Pre/post	Reference: No *Reason for selection:* No	Gastrointestinal discomfort	No data presented
Cameron et al. [46]	25 elite male rugby players (21.6 ± 2.6 yrs)	25-min warm-up followed by 9 min of high-intensity rugby-specific training followed by a rugby-specific repeated-sprint test	9 × 100 mm VAS. Anchored at each end with *no symptom* on the left-hand side and *severe symptom* on the right-hand side	30-min	Reference: No *Reason for selection:* No	Nausea, flatulence, stomach cramping, belching, stomach-ache, bowel urgency, diarrhea, vomiting, stomach bloating	*Statistical method:* McNemar's test for presence/absence of symptom & Wilcoxon's matched-pairs signed-ranks test for the degree of the symptom reported. *Data reported:* Mean ± SEM
Costa et al. [47]	25 runners (15 M, 10 F, 35 (32–38) yrs)	2 h running exercise at 60% $\dot{V}$ O_2max_ and a 1 h distance test	A 10-point Likert type. 0 = no symptom to 10 indicative of extreme symptom (5 indicative of severe symptom)	20-min	Reference: Costa et al. 2016 *Reason for selection:* No	Projectile vomiting, regurgitation, urge to regurgitate, gastric bloating, belching, stomach pain, and heartburn/gastric acidosis, flatulence including lower abdominal bloating, urge to defecate, abdominal pain, abnormal defecation, nausea, dizziness, and stitch	*Statistical method:* Kruskal–Wallis one-way and Mann–Whitney *U* test *Data reported:* Median & IQR
Campagnolo et al. [48]	10 endurance trained males (25 ± 5 yrs)	60-min cycle	100 mm visual analog scale, with 0 mm representing “not at all” and 100-mm representing “extremely”	Pre/post	Reference: No *Reason for selection:* No	Bloating	*Statistical method:* Two-way repeated-measures ANOVA (trial × time) *Data reported:* Mean ± SD
Chantler et al. [49]	19 elite male academy rugby players (20 ± 1 yrs)	Rugby training session	0–5 scale (0 = nothing, 1 = just noticeable, not painful, 2 = noticeable, 3 = quite noticeable, mildly painful or disruptive, 4 = painful, quite disruptive, but does not prevent any day-to-day activities,5 = painful, may need medication and disrupts the flow of daily activities)	Pre/post	Reference: McLean et al. [50]. *Reason for selection:* The scale of symptoms was modified to align with the other wellness monitoring done with the players	GIS; range of thirteen standard symptoms	*Statistical method:* Kruskal–Wallis one-way and Mann–Whitney *U* test *Data reported:* median & IQR & frequency of the number of players
Clayton et al. [51]	23 elite male professional footballers (24.3 ± 2.8 yrs)	6 competitive matches	0 to 9 (0 = “no problem at all”, 9 = “worst it's ever been”)	Pre/during/post	Reference: Pfeiffer et al. [4] *Reason for selection:* “standardized questionnaire previously demonstrated to detect changes in GIS in athletes”	Reflux/heartburn, belching, bloating, stomach cramps/pain, nausea, vomiting, intestinal/lower abdominal cramps, flatulence, urge to defecate, side ache/stitch, loose stool, diarrhea, intestinal bleeding, dizziness, headache, muscle cramp, urge to urinate	*Statistical method*: Paired sample t-tests *Data reported*: individual player responses to GIS per day (normalized over time)
Convit et al. [52]	44 ultramarathon runners (40 ± 8 yrs)	56 km trail ultramarathon event	VAS scale (10-point rating, each point indicative of10 mm	Pre/post	Reference: Gaskell et al. [19] *Reason for selection:* Validated and reliable	Belching, heartburn, bloating, stomach pain, urge to regurgitate, regurgitation, projectile vomiting, flatulence, bloating, urge to defecate, intestinal pain, abnormal ­defecation, nausea, dizziness, abdominal stitch	*Statistical method*: Mann–Whitney U-test *Data reported*: accumulative score with median (range) and incidence (%)
Convit et al. [53]	11female runners (33 ± 9 yrs)	10 km running TT in 34 °C and 60% relative humidity	11-point Likert rating scale (0–10)	Pre, during and post exercise	Reference: Gaskell et al. [19] *Reason for selection:* No	Belching, heartburn, bloating, stomach pain, urge to regurgitate, regurgitation, projectile vomiting, flatulence, bloating, urge to defecate, intestinal pain, abnormal ­defecation, nausea, dizziness, abdominal stitch	*Statistical method*: linear mixed model *Data reported*: median (IQR)
Costa et al. [21]	Multi-stage (*n* = 54) and continuous (*n* = 22) ultramarathons	Multi-stage and 24 h continuous ultramarathon	A ≥ 50 mm on a standard 100 mm VAS, with 0 mm indicative of no symptoms to 100 mm indicative of extreme symptoms. Severity of GIS was classified as low, moderate, and high based on the incidence of GIS reported (1 GIS, 2–3 GIS, and ≥ 4 GIS; respectively)	Post	Reference: No *Reason for selection:* No	Not stated	*Statistical method:* Kruskal–Wallis one-way and Mann–Whitney *U* test *Data reported:* Percentage of participants reporting GIS
Costa et al. [54]	11 male runners (34 ± 11 yrs)	2 h run 70% $\dot{V}$ O_2max_	VAS: 1–4 indicative of mild GIS (i.e. sensation of GIS, but not substantial enough to interfere with exercise workload) and increasing in magnitude, 5–9 indicative of severe GIS	15-min	Reference: No *Reason for selection:* No	Nausea, flatulence, stomach cramping, belching, stomach-ache, bowel urgency, diarrhea, vomiting, stomach bloating	*Statistical method:* One- or two-way repeated-measures ANOVA *Data reported:* The GIS incidence = descriptive percentage, GIS severity & feeding tolerance markers = accumulative score & individual participant range
Costa et al. [55]	20 endurance running trained males age (32 ± 8 yrs)	2 h SS run	VAS	20-min	Reference: Gaskell et al. [19] *Reason for selection:* No	Belching, Heartburn, Bloating, Stomach pain, Urge to regurgitate, Regurgitation, Projectile vomiting, Flatulence, bloating, Urge to defecate, Intestinal pain, Abnormal ­defecation, Nausea, Dizziness, Abdominal stitch	*Statistical method:* Friedman test *Data reported*: Mean of summative accumulation and range of participants reporting incidence
Costa et al. [56]	11 male endurance runners (34 ± 11 yrs)	2 h run at 70% of $\dot{V}$ O_2max_	mVAS (10-point rating scale, each point indicative of 10 mm)	15-min	Reference: No *Reason for selection:* No	Belching, Heartburn, Bloating, Stomach pain, Urge to regurgitate, Regurgitation, Projectile vomiting, Flatulence, bloating, Urge to defecate, Intestinal pain, Abnormal ­defecation, Nausea, Dizziness, Abdominal stitch	*Statistical method:* Two-way repeated-measures ANOVA *Data reported:* overall participant summative accumulation of measured time periods & individual range of those participants reporting symptoms
Costa et al. [57]	28 endurance athletes	2 h running at 55 % of $\dot{V}$ O_2max_ or cycling at 55% of maximal aerobic power	mVAS (10-point rating scale, each point indicative of 10 mm)	10 mins	Reference: No *Reason for selection:* No	Belching, Heartburn, Bloating, Stomach pain, Urge to regurgitate, Regurgitation, Projectile vomiting, Flatulence, bloating, Urge to defecate, Intestinal pain, Abnormal ­defecation, Nausea, Dizziness, Abdominal stitch	*Statistical method:* Two-way repeated-measures ANOVA *Data reported:* overall participant summative accumulation of measured time periods & individual range of those participants reporting symptoms
Cunniffe et al. [58]	10 healthy well-trained men (23 ± 4 yrs)	10 maximal cycle sprints (with 30-second rest intervals) and cycle TTE at 100% of individual PP	GI discomfort were taken using a Likert (ranging from 6 = none to 20 = very, very strong) scale	15-min	Reference: No *Reason for selection:* No	Bloatedness, nausea, and reflux	*Statistical method:* Wilcoxon signed rank test *Data reported*: Mean ± SD
Dobbin et al. [59]	15 academy rugby league players	two bouts of rugby activity	A 0–100 VAS (0 = none– 100 = severe)	Pre/post	Reference: No *Reason for selection:* No	Gut discomfort	No data provided
Dudar et al. [60]	14 Trained endurance runners (8 M, 6 F)	IET at 55, 65, and 75% $\dot{V}$ O_2peak_ and a 5-km TT	VAS scale (“0 mm” = no GID and “100 mm” = extreme GID)	Post	Reference: Jeukendrup et al., 2000 *Reason for selection:* No	Nausea, stomach-ache, urge to vomit, vomiting and intestinal cramps. Less severe GI symptoms included eructation, flatulence, urge to defecate, heartburn and abdominal pressure (bloating)	*Statistical method:* A one-way repeated measures ANOVA *Data reported*: Mean ± SD
Durkalec-Michalski et al. [61]	19 CrossFit athletes (37 ± 8 yrs)	6 bouts of 15 s WAnT	NRS ranged between 0 and 10, where 0 reflected no GI distress at all and 10 being the most severe GI distress imaginable. GIS were considered severe when a score ≥ 5	Post	Reference: Jeukendrup et al. 2000 *Reason for selection:* No	Flatulence, urge to defecate, belching or diarrhea, as well as total GIS	*Statistical method*: Friedman's ANOVA *Data reported*: Median ± IQR
Durkalec-Michalski et al. [62]	30 CrossFit athletes (26 M; 31.9 ± 3.2 yrs, 14 F; 21.9 ± 5 yrs)	The exercise battery lasted 7 min with two 30-s Wingate Anaerobic Tests and exercises performed for 3 min: a) Wall Balls and b) Burpees	0 to 10, where 0 reflects no GI distress at all and 10 reflects the most severe GI distress imaginable	Post	Reference: Jeukendrup et al. 2000 *Reason for selection:* No	Flatulence, urge to defecate, belching or diarrhea, as well as total GIS	*Statistical method*: Friedman's ANOVA *Data reported*: Median ± IQR
Edwards et al. [63]	*n* = 10 (5 F, 5 M) *n* = 11 (3 F, 8 M)	3 cycling (60 min at 40%, 60% and 80% $\dot{V}$ O_2max_ or one running and 1 cycling trial (45 min at 70% $\dot{V}$ O_2max_	10-point scale ranging from 0 (“no problem at all”) to 9 (“the worst it has ever been”)	Didn't state	Reference: adapted from Pfeiffer et al. 2009 *Reason for selection:* No	Upper abdominal, lower abdominal and other symptoms	No data provided
Ehlert & Wilson [64]	82 runners (43 M, 39 F, 47 ± 19 yrs	During run training	GIS were rated on a 0–10 scale (virtual)	Post exercise	Reference: Wilson et al. 2017 *Reason for selection:* No	Nausea, reflux/regurgitation, bloating, and stomach fullness, abdominal cramps, gas/flatulence, and urge to defecate	*Statistical method*: Spearman's *ρ* coefficients *Data reported*: median (IQR)
Etxebarria et al. 65	12 well-trained male runners (37 ± 7 yrs)	15 min at 60% $\dot{V}$ O_2peak_, 15 min at 75% $\dot{V}$ O_2peak_, followed by 8 × 1-min high-intensity efforts	frequency of GI discomfort from 0 (never) to 4 (all the time) and adverse symptoms	Pre and 72 h post	Reference: West et al. 2013 *Reason for selection:* No	Not stated	*Statistical method*: Did not state specific analysis *Data reported*: Mean ± SD
Flood et al. [66]	14 road cyclists (7 M; 27 ± 8 yrs, 7 F; 23 ± 2 yrs)	90 min cycle (45% $\dot{V}$ O_2pmax_), 15-min TT in hot (32 °C, 70% RH)	Completed a gut questionnaire regarding the presence of GI problems at that moment	30-min	Reference: Referenced Gaskell et al. [19] *Reason for selection:* No	Belching, heart burn Bloating, upper abdominal pain, urge to vomit, vomit, flatulence, urge to defecate Abdominal pain, nausea dizziness, abdominal stitch	*Statistical method*: Friedman's test, & main effects explored using Dunn's test *Data reported*: Mean (range within an individual participant)
Geaney et al. [67]	15 males (35.2 ± 7.4 yrs)	60 min run at 60% $\dot{V}$ O_2peak_, and a TTE	0–10 questionnaire	During/post	Reference: Wilson et al. 2017 *Reason for selection:* No	Nausea, belching, regurgitation/reflux, stomach fullness, bloating, side stitching, abdominal cramps, gas, urge to defecate	*Statistical method*: Wilcoxon signed-rank test *Data reported*: Summative accumulation scores
Gaskell et al. [68]	16 runners (8 M, 8 F; 38 ± 11 yrs)	1 h, 2 h and 3 h run at 60% $\dot{V}$ O_2max_	1–4 indicative of mild GIS (i.e. sensation of GIS, but not substantial enough to interfere with exercise workload) and increasing in magnitude, 5–9 indicative of severe GIS (i.e. GIS substantial enough to interfere with exercise workload), and 10 indicative of extremely severe GIS warranting exercise cessation. A rating of 0 was recorded if no specific GIS was reported	Pre/post	Reference: Scale from Gaskell et al. [19] *Reason for selection:* No	Projectile vomiting, regurgitation, urge to regurgitate, gastric bloating, belching, stomach pain, and heartburn/gastric acidosis, flatulence including lower abdominal bloating, urge to defecate, abdominal pain, abnormal defecation, nausea, dizziness, and stitch	*Statistical method*: two-way repeated-measures ANOVA *Data reported*: Summative accumulation
Gaskell et al. [22]	16 endurance runners (43 ± 9 yrs)	3 h run at 60% $\dot{V}$ O_2max_	(mVAS) GIS assessment tool: 10-point rating scale	15-min	Reference: Referenced Gaskell et al. [19] *Reason for selection:* No	Belching, heartburn, upper abdominal bloating, upper abdominal pain, urge to regurgitate, regurgitation, flatulence, lower abdominal bloating, urge to defecate, intestinal pain, abnormal stools, nausea, dizziness, abdominal stitch	*Statistical method*: non-parametric equivalents, such as Wilcoxon signed-rank test *Data reported*: Summative accumulation
Gaskell et al. [19]	31 recreational endurance runners (36 ± 8 yrs)	2 h run 70% $\dot{V}$ O_2max_ temperate, 2 h run at 60% $\dot{V}$ O_2max_ in hot (35.1 °C), 2 h run at 60% $\dot{V}$ O_2max_ in temperate	mVAS scale (incidence of severe symptoms (≥5 on the 0–10)	Pre/post	Reference: No *Reason for selection:* No	Belching, heartburn, bloating, stomach pain, urge to regurgitate, regurgitation, projectile vomiting, flatulence, bloating, urge to defecate, intestinal pain, abnormal ­defecation, nausea, dizziness, abdominal stitch	*Statistical method*: Wilcoxon signed-rank test *Data reported*: Accumulative score and individual participant range for GIS
Gaskell et al. [69]	18 endurance runners (35 ± 8 yrs)	2 h of running at 60 % *V̇*O_2max_ in 35 °C	mVAS: 10-point rating scale, each point indicative of 10 mm	30-min	Reference: Referenced Gaskell et al. [19] *Reason for selection:* No	Belching, heartburn, upper abdominal bloating, upper abdominal pain, urge to regurgitate, regurgitation, flatulence, lower abdominal bloating, urge to defecate, intestinal pain, abnormal stools, nausea, dizziness, abdominal stitch	*Statistical method*: non-parametric equivalents, such as Wilcoxon signed-rank test *Data reported*: Summative accumulation
Gaskell et al. [70]	16 endurance runners (8 M, 8 F, 38 ± 11 yrs)	2 h of running at 60 % *V̇*O_2max_ in 35 and 22 °C ambient conditions	mVAS: 10-point rating scale, each point indicative of 10 mm	Pre/during/post	Reference: Gaskell et al. [19] *Reason for selection:* No	Projectile vomiting, regurgitation, urge to regurgitate, gastric bloating, belching, stomach pain, and heartburn, flatulence including lower-abdominal bloating, urge to defecate, abdominal pain, defecation with or without abnormalities including loose water stools, diarrhea and blood in stools, nausea, dizziness, and acute transient abdominal pain (stitch)	*Statistical method*: two-way (trial*time) repeated-measures ANOVA *Data reported*: accumulative score (total and corrected) and individual participant range
Glace et al. [71]	19 runners	160-km ultrarun	Investigators interviewed each subject as to the occurrence of any GI distress. Runners were asked to describe symptoms, approximately when they occurred, and duration	First half, second half	Reference: No *Reason for selection:* No	Such as nausea, vomiting, or diarrhea	*Statistical method*: ANOVA *Data reported:* Data reported ± SEM & number of participant reported
Goedecke et al. [72]	8 endurance-trained cyclists (21 ± 3 yrs)	60-min intervals, followed immediately by a 200 kJ TT	The severity of the symptoms was rated on a scale of 0 to 3. 0 = no symptoms, 3 = symptoms severe enough to interfere with exercise performance	30-min	Reference: No *Reason for selection:* No	Nausea, vomiting, stomach cramps, bloating, and diarrhea	*Statistical method*: ANOVA *Data reported*: Mean ± SD
Gurton et al. [73]	8 male cyclists (23 ± 7 yrs)	4 × 4-km cycling TT'	VAS (0 mm = “no symptom”; 100 mm = “severest symptom”)	baseline, pre-warm up, pre-TT, post-TT	Reference: No *Reason for selection:* No	Belching, vomiting, flatulence, diarrhea, belching, abdominal discomfort, gut fullness, bowel urgency rating	*Statistical method*: Friedman's two-way ANOVA *Data reported:* Symptom severity score (on a scale of 0 to 100)
Gough et al. [74]	11 recreationally trained male cyclists (28 ± 6 yrs)	4 km cycling TT in the heat	VAS - 20 cm horizontal line that was anchored with “no symptom” to the left and “severe discomfort” to the right	15-min	Reference: Miller et al., 2016 *Reason for selection:* No	Belching, vomiting, flatulence, diarrhea, belching, abdominal discomfort, gut fullness, bowel urgency rating	*Statistical method*: Wilcoxon matched-paired signed-rank test *Data reported:* median & z values. Symptom severity score (on a scale of 0–20)
Gough & Sparks [75]	10 well-trained cyclists (31 ± 8 yrs)	2 × 4 km cycling TT	VAS with 0 and 10, with 0 representing no symptoms and 10 representing most severe symptoms	Pre/during/post	Reference: No *Reason for selection:* No	Nausea, flatulence, stomach cramping, belching, stomach ache, bowel urgency, diarrhea, vomiting and stomach bloating	*Statistical method*: Wilcoxon test *Data reported:* mean ± SD
Guillochon & Rowlands [76]	12 well-trained male cyclists (33.6 ± 9.4 yrs)	4 trials comprising a 140-min race simulation, followed by a double-blind slow-ramp to exhaustion (0.333 W·s-1)	Did not state	20-min	Reference: No *Reason for selection:* No	Nausea, stomach fullness, abdominal cramping	*Statistical method*: grand mean centered x-axis variable (time) was added for linear modeling *Data reported*: Reported mean effect ± 90% CI
Haakonssen et al. [25]	32 well-trained female cyclists (24.3 ± 4.1 yrs)	90-min cycle session (80-min at 60% maximal aerobic power and 10-min TT	VAS: how comfortable does your stomach feel at the moment? according to a five point scale (1 = very comfortable; 2 = comfortable; 3 = average comfort; 4 = uncomfortable; 5 = very uncomfortable)	Before pretrial meal, 30/60 min after starting pretrial meal, pre and post exercise	Reference: No *Reason for selection:* No	Nausea, bloating and gut pain	*Statistical method*: Fishers exact test *Data reported*: Change scores (pre-post trial)
Hansen et al. [77]	28 runners (22 M, 6 F; 34/42 ± 10/8 yrs)	Marathon race	10-point scale ranging from 0, no problem at all, to 9, the worst it has ever been	15-min	Reference: Pfeiffer et al. 2009	Reflux, heartburn, nausea, bloating, vomiting, abdominal pain, urge to defecate, and diarrhea—as well as such systemic symptoms as headache, dizziness, urge to urinate, and muscle cramps	*Statistical method*: Wilcoxon's signed-rank tests *Data reported:* Mean (median*)*
Hart et al. [78]	30 resistance-trained (15 M; 24 ± 4, 15 F; 24 ± 4 yrs)	The RE protocol utilized a load of 70% 1RM for 4 sets of 10 reps (squat, seated shoulder press, deadlift, bent-over row, leg press)	10-point (0 = no problem at all to 9 = the worst it has ever been). Overall symptoms were classified as a rating of ≥ 2 and a moderate symptom was defined as a rating of ≥ 4	Pre/post	Reference: Pfeiffer et al. 2009 & Pugh et al. 2020 *Reason for selection:* No	Reflux, heartburn, bloating, cramps, vomiting, nausea, intestinal cramps, flatulence, urge to defecate, left/right abdominal pain, loose stool, diarrhea	*Statistical method*: Pearson product moment correlation *Data reported*: Summative mean ± SD
Hall et al. [24]	10 well-trained males (30 ± 8 yrs)	2.5 h cycle, 4 h recovery period and a performance time trial (total work, 7 kJ·kg^−1^).	4-point scale (0 = Hardly any complaint, 1 = moderate complaint, 3 = severe complaint, 4 = very severe complaint)	20-min	Reference: Peters et al. 1999. *Reason for selection*: No	Bloating, belching, nausea, vomiting, abdominal cramps, side stich	*Statistical method*: ANOVA by general linear modeling No data reported
Henningsen et al. [79]	132 endurance-trained individuals (97 M, 35 F, 32.1 ± 7.7 yrs)	2 h of running exercise in temperate (21.2–30.0 °C) to hot (35.0–37.2 °C) ambient conditions	exercise-specific GIS assessment tool (mVAS)	Pre, during and post exercise	Reference: Costa et al., 2016, Gaskell et al. [19] *Reason for selection:* No	Belching, gastric bloating, Upper abdominal pain, urge to regurgitate, and/or regurgitation), lower-gis (i.e. Intestinal: flatulence, lower abdominal bloating, lower abdominal pain, urge to defecate, and/or abnormal defecation), and others (i.e. Acute transient abdominal Pain (stitch), dizziness, and nausea	*Statistical method*: Spearman's rank correlation coefficients and Simple linear regression analyzes *Data reported*: mean ± SD or mean and 95% confidence interval (CI), as indicated; and total accumulative score and 95% CI for Ex-GIS
Jardine et al. [80]	13 endurance runners (33 ± 7 yrs)	Half-marathon TT	0–10 scale (5 classified as severe)	Pre/during/post	Reference: No *Reason for selection:* No	18 questions for “upper”, “lower,” and “other” symptoms	*Statistical method*: Linear mixed model *Data reported*: Mean ± SD
Jones et al. [81]	20 recreationally active males (24 ± 3 yrs)	180 min cycle at LT1 and a TTE at 150% LT1	0–10 scale (0, 5 and 10 indicating no discomfort at all, moderate and unbearable discomfort, respectively)	During/post	Reference: Wilson et al. 2017 *Reason for selection:* No	Nausea, reflux, stomach fullness, abdominal cramps, flatulence and urge to defecate	*Statistical method*: Repeated-measures ANOVA *Data reported*: Mean ± SD
Jonvik et al. [82]	16 well-trained male athletes (28 ± 7 yrs)	60 min at 70% Wmax	VAS scale The 16-item Questionnaire. 50% on the scale is defined as a severe symptom.	Pre/Post	Reference: Jeukendrup et al. 2000 *Reason for selection:* No	Did not specify symptoms	*Statistical method*: Repeated-measures ANOVA *Data reported*: Mean ± SD
Jentjens et al. [23]	9 trained cyclists (24.4 ± 2.6 yrs)	90 min at 55% max power output	10-point scale (1 = not at all, 10 = very, very much)	During/post	Reference: Jeukendrup et al. 2000 for severity classification *Reason for selection:* No	Stomach problems, GI cramping, bloated feeling, diarrhea, nausea, dizziness, headache, belching, vomiting, and urge to urinate/defecate	*Statistical method*: Not stated specific analysis *Data reported*: Number of reported incidences
Jeukendrup et al. [9]	29 athletes (33 ± 6 yrs)	Long-distance triathlon	Only information provided was that symptoms were severe when a score of 5 or higher out of 10 was given	During/post	Reference: No *Reason for selection:* No	Severe symptoms: nausea, urge to vomit, vomiting, stomach-ache, intestinal cramps, eructation, flatulence, urge to defecate, heart burn, abdominal pressure (bloating). Nausea, stomachache, intestinal cramps, urge to vomit were non-severe symptoms when a score < 5 was given	*Statistical method*: Spearman Rank Correlation test with other markers *Data reported*: Number of reported symptoms and percentage
Kahle et al. [83]	11 endurance-trained individuals (5 M, 6 F; 24.6 ± 0.5 yrs)	20 min of submaximal cycling exercise	0–10 scale,0 = GI distress at all and 10 being the most severe GI distress imaginable	The nature of the symptom monitoring was open-ended and did not involve specific questions at set time intervals	Reference: Dworkin et al. 2005, Von Korff et al. 2000 *Reason for selection:* Verbal numeric rating scales are common and valid for measuring patient-assessed global pain and have also been validated for use in evaluating global symptom severity in patients with IBS	Nausea, vomiting, diarrhea, bloating, cramping, light-headedness, and thirst	*Statistical method*: Single-sided non-parametric Cohen *κ* coefficients *Data reported*: Mean ± SEM.
Kelly et al. [84]	40 runners (M 19, F 21; 44.9 ± 10.6 yrs)	Boston Marathon	10-point scale (0 = no problems at all and 9 = the worst it has ever been)	Pre/post	Reference: Pfeiffer et al., 2009, Pfeiffer et al., 2012 *Reason for selection:* No	Reflux/heart burn, belching, bloating, stomach pain/ Cramps, vomiting, nausea, intestinal cramps, flatulence, urge to defecate, left and right Abdominal pain/stitch, loose stool, diarrhea	*Statistical method*: 1 × 3 repeated measures ANOVA *Data reported*: mean ± SD
King et al. [85]	18 highly trained distance runners	35-km run including a 7 km TT	10-point rating scale. 1–4 indicative of mild GIS (i.e. the sensation of GIS, but not substantial enough to interfere with exercise workload) and increasing in magnitude, 5–9 indicative of severe GIS (i.e. GIS substantial enough to interfere with exercise workload), and 10 indicative of extremely severe GIS	7 occasions throughout	Reference: Scale from Gaskell et al. [19] *Reason for selection:* No	Projectile vomiting, regurgitation, urge to regurgitate, gastric bloating, belching, stomach pain, and heartburn/gastric acidosis, flatulence including lower abdominal bloating, urge to defecate, abdominal pain, abnormal defecation, nausea, dizziness, and stitch	*Statistical method*: general linear mixed model *Data reported*: Mean ± SD
Kuru et al. [86]	20 male soccer players (20.9 ± 2.3 yrs)	Running-based anaerobic sprint test	5-point Likert-type scale, ranging from 0 (indicating no problem) to 4 (indicating a very severe problem)	Pre/during/post	Reference: Adam et al. 2005 *Reason for selection:* using a validated questionnaire	nausea, vomiting, bloating, abdominal cramps, early satiety, heartburn, sickness, loss of appetite, retrosternal discomfort, and upper abdominal pain	*Statistical method*: Repeated measures ANOVA *Data reported*: mean ± SD
Lambert et al. [87]	7 trained runners (5 F, 2 M; 22 ± 2 yrs)	6 × 90 min runs (65 % $\dot{V}$ O_2max_)	Stomach comfort (1—4 scale). 1 = very comfortable to 4 = very uncomfortable	10-min	Reference: No *Reason for selection:* No	Stomach comfort	*Statistical method*: Friedman non-parametric test *Data reported*: median & range
Lee et al. [88]	25 male ARF players (15.7 ± 0.3 yrs)	ARF 7-day national tournament	4 point scale (0 = Hardly any complaint, 1 = moderate complaint, 3 = severe complaint, 4 = very severe complaint)	Not stated	Reference: Based on Lambert et al. 2001 *Reason for selection* No	Belching, nausea, vomiting, abdominal pain, side stitch, urge to defecate, flatulence, and diarrhea	*Statistical method*: Mann-Whitney U-test *Data reported*: Median & range
Lambert et al. [89]	8 runners (22.5 ± 2.5 yrs)	3 × 60-min treadmill runs at 70% $\dot{V}$ O_2max_	Placed a mark on a 133-mm scale indicating their level of comfort	10-min	Reference: No *Reason for selection:* No	Fullness, heartburn, nausea, abdominal cramps, urge to defecate	*Statistical method*: Friedman non-parametric test. *Data reported*: median & range
Lambert et al. [90]	17 runners (13 M, 4 F; 27 ± 1 yrs)	60-min treadmill runs at 60% $\dot{V}$ O_2max_	VAS (10- mm lines)	Pre/post	Reference: No *Reason for selection:* No	Heartburn, nausea, side ache, diarrhea	*Statistical method*: Friedman non-parametric test. *Data reported*: median & range
Leach et al. [91]	10 trained male cyclists (31.1 ± 9 yrs)	16.1-km cycling TT	10-point VAS. The scale was anhored with 0 (no symptom) on the left to 10 (severe symptom)	Not stated	Reference: No *Reason for selection:* No	Diarrhea, stomach cramps, nausea, stomach-ache, bloating, flatulence, bowel urgency, belching, and vomiting	*Statistical method*: GIS scores were aggregated to determine the total number of symptoms experienced for each participant. Total GIS was analyzed using paired t-tests *Data reported*: mean ± SD
Lennon et al. [92]	16 runners (8 M, 8 F, 32.7 ± 8 yrs)	Run at 65–70% of VO2max	10-point scale, ranging from 0 or “no problem at all” to 9 or “the worst it has ever been”	During/post	Reference: Pfeiffer et al., 2012 *Reason for selection: “*using a questionnaire developed by Pfeiffer et al”	Reflux/heartburn, belching, bloating, stomach pain/cramps, Vomiting, nausea, intestinal/lower abdominal cramps, side ache/stitch, Flatulence, urge to defecate, diarrhea, intestinal bleeding)	*Statistical method*: repeated measures mixed-effect model *Data reported*: Cumulative abdominal symptom score
Martin et al. [93]	11 female cross-fit athletes (29 ± 4 yrs)	2 × 10 maximal strokes prior to the 2-km TT	Not stated	Not stated	Reference: No *Reason for selection:* No	Nausea, diarrhea, stomach bloating, belching, bowel urgency, stomach cramping	*Statistical method*: weighted cases chi squared test (χ2). aggregated GIS were assessed using Spearman's correlation coefficients *Data reported*: Individual GIS scores
Martinez et al. [94]	20 endurance athletes (35 ± 10 yrs)	2 h of running (60 % ˙VO2max), followed by a 1 h self-paced distance test	modified visual analog scale (mVAS)	Pre/during/post	Reference: Gaskell et al., [19] *Reason for selection:* No	Belching, heartburn, bloating, stomach pain, urge to regurgitate, regurgitation, projectile vomiting, flatulence, bloating, urge to defecate, intestinal pain, abnormal ­defecation, nausea, dizziness, abdominal stitch	*Statistical method*: ANOVA *Data reported*: accu­ mulative score or individual participant range
Martinez et al. [95]	53 endurance athletes (37 ± 11 and 34 ± 10 yrs)	2 h of running (60 % ˙VO2max), followed by a 1 h self-paced distance test	modified visual analog scale (mVAS)	Pre/during/post	Reference: Gaskell et al., [19] *Reason for selection:* No	Belching, heartburn, bloating, stomach pain, urge to regurgitate, regurgitation, projectile vomiting, flatulence, bloating, urge to defecate, intestinal pain, abnormal ­defecation, nausea, dizziness, abdominal stitch	*Statistical method*: ANOVA *Data reported*: accumulative score or individual participant range
Mooren et al. [96]	19 males (26.5 ± 4.7 yrs)	1 h run at 60-80% $\dot{V}$ O_2max_.	Self-developed 10-point likert scale	During and up to 3 h post	Reference: No *Reason for selection:* No	Covering 19 items, such as stomach cramps, nausea and heartburn	*Statistical method*: Repeated measures ANOVA *Data reported*: Not reported
Morrison et al. [97]	Trained (*n =* 7) and untrained (*n =* 7) males. (22 ± 3 yrs)	15-min cycle at 50% HRR before and after 60-min run (30-min at 80% HRR then 30-min distance trial	0 (no symptoms), to 10 (severely uncomfortable)	15-min	Reference: No *Reason for selection:* No	Heartburn, nausea, abdominal cramps, bloating, and urge to have a bowel movement	*Statistical method*: Three-way repeated measures ANOVA *Data reported*: Mean ± SD
Miall et al. [98]	18 recreational runners (35 ± 8 yrs)	2 h SS run at 60% $\dot{V}$ O_2max_ & 1 h running effort bout.	10-point Likert type. 0 = no symptom to 10 indicative of extreme symptom (5 indicative of severe symptom)	10-min	Reference: Costa et al. 2016 *Reason for selection:* No	Projectile vomiting, regurgitation, urge to regurgitate, gastric bloating, belching, stomach pain, and heartburn/gastric acidosis, flatulence including lower abdominal bloating, urge to defecate, abdominal pain, abnormal defecation, nausea, dizziness, and stitch	*Statistical method*: Friedman non-parametric test. *Data reported*: Mean ± SEM
Mazur-Kurach et al. [99]	26 road cyclists 23.25/21.28 yrs	Anaerobic Wingate test and aerobic test using a cycle ergometer	(0 = none, 1 = mild, 2 = moderate, 3 = severe)	Not stated	Reference: No *Reason for selection:* No	Nausea, vomiting, diarrhea, abdominal pain, heartburn, and gastrointestinal bleeding	Data not reported
McCubbin et al. [100]	9 male endurance runners (36 ± 5 yrs)	3 h of SS running at 60% $\dot{V}$ O_2max_, followed by a TTE test.	0–11 scale incidence of severe symptoms (≥5 on the 0–10)	15-min	Reference: No *Reason for selection:* No	Nausea, belching, bloating, pain, regurgitation, flatulence, urge to defecate, and diarrhea	*Statistical method*: Wilcoxon matched-pairs signed-rank test *Data reported*: Mean (range within individual participants)
McCarthy et al. [101]	23 trained cyclists (31 ± 9 yrs)	20 min cycling TT	0–10 scale (0 was no symptom present, 1–4 mild symptoms, 5–9 severe symptoms, and 10 extremely severe symptoms)	During	Reference: Gaskell et al. [19] *Reason for selection:* “a validated gastrointestinal symptom questionnaire for sports nutrition exercise trials to assess perceptions experienced during”	Belching, heartburn, stomach Bloating, upper abdominal pain, urge to regurgitate, and/or Actual regurgitation, flatulence, Lower abdominal bloating, lower abdominal pain, urge to Defecate, and abnormal defecation (e.g. loose watery stools, diarrhea, and/or fecal blood loss)	*Statistical method*: Wilcoxon test *Data reported*: total severity
Ma et al. [102]	16 young men	60 min cycle at 70% HRmax	7-point scale, where 1 = means “absolutely no” and 7 = “absolutely yes”	Post	Reference: No *Reason for selection:* No	Not provided	No data provided
Miller et al. [44]	11 participants (24 ± 6 yrs)	RSA cycling protocol (10 × 6 s sprints with 60 seconds recovery	100 mm VAS anchored at each end starting with *no symptom* on the left and *severe symptom* on the right.	Pre/post drink	Reference: Carr et al. 2011 *Reason for selection:* No	Nausea, flatulence, stomach cramping, belching, stomach ache, bowel urgency, diarrhea, vomiting, and stomach bloating	*Statistical method*: Paired samples t-test *Data reported*: mean ± SD
Moncada-Jiménez et al. [103]	11 males (37 ± 5 yrs)	laboratory-based duathlon (5-km run, 30-km cycling, 10-m run)	mVAS to determine anatomical position	Pre/post	Reference: Morton & Callister, 2002 *Reason for selection:* No	Belching, bloating, diarrhea, dizziness, flatulence/gas, headache, heartburn, intestinal cramps, muscle cramps, nausea, side-pain/side stitch, stomach cramps, stomach upset, urge to defecate, urge to urinate, urge to vomit, vomiting, and/or other	*Statistical method*: Chi2 (χ2) *Data reported*: Frequency of participants with GIS
Naderi et al. [104]	11 junior male professional taekwondo athletes (16 ± 0.8 yrs)	The kicking test was repeated 5 times, separated by 45 min of rest, simulating a typical taekwondo competition day	A validated gastrointestinal questionnaire was also used to assess GI distress immediately after each test	Post	Reference: Pfeiffer, et al. 2009 *Reason for selection:* No	Reflux/heartburn, severe belching, bloating, stomach pain/cramps, vomiting, and nausea	Data not reported as one participant experienced symptoms
Nomura et al. [105]	11 healthy males (23 ± 1 yrs)	60 min run on a treadmill at 70% maximal running velocity	1–10 cm visual analog scale (VAS). Severity was rated as mild (1–3), severe (4–5), or very severe (7–10)	Pre/post	Reference: Mckenna et al. 2022 *Reason for selection:* No	Feelings of hunger, fullness, bloating, abdominal Pain, nausea, and upset stomach	*Statistical method*: ANOVA *Data reported*: mean ± SD
Ogden et al. [106]	10 healthy males (29 ± 7 yrs)	Running in the heat (40 °C and 40% RH) at LT to volitional exhaustion.	GIS are presented as the incidence (%) and accumulated severity of symptoms	10-min	Reference: Gaskell et al. [19] *Reason for selection:* following previous guidance	Belching, heartburn, bloating, stomach pain, urge to regurgitate, regurgitation, projectile vomiting, flatulence, bloating, urge to defecate, intestinal pain, abnormal ­defecation, nausea, dizziness, abdominal stitch	*Statistical method*: Two-way repeated measures ANOVA *Data reported*: accumulated mean ± range of reported symptoms ≥ 1 (Gaskell et al., 2019)
Opheim & Rankin [107]	19 healthy males (23 ± 3 yrs)	RST consisting of 15 30-m maximal effort sprints on 35-sec intervals	0 (none) to 5 (severe) to indicate magnitude of the symptom.	Pre/mid/post	Reference: No *Reason for selection:* No	Intestinal cramping, diarrhea, nausea, flatulence, and stomach discomfort	*Statistical method*: repeated measures of ANOVA *Data reported:* mean ± SD
Oosthuyse et al. [108]	8 trained, male cyclists (38.9 ± 6.3 yrs)	2 h of exercise at 60 %W_max_ followed by a 16-km TT	10-point scale, where corresponds to nothing, 1 very weak/mild; 2 weak; 3 moderate; 5 strong; 7 very strong; and 10 extremely strong	30-min	Reference: Rowlands et al. 2008 *Reason for selection:* No	Bloating, nausea, stomach cramps	*Statistical method*: Inferential analysis *Data reported*: mean ± SD
Oosthuyse et al. [38]	9 trained male (38 ± 7 yrs)	2 h SS (exercise (60%W_max_) followed by a 16 km TT	10-point scale (0 = nothing, 1 = very weak, mild; 2 = weak; 3 = moderate, 5 = strong; 7 = very strong, and 10 = extremely strong)	30-min	Reference: Rowlands et al. 2008 *Reason for selection:* No	Thirst, bloating, nausea, stomach cramps and sweetness of the drink	*Statistical method*: Inferential analysis *Data reported*: mean ± SD
Pugh et al. [109]	24 runners (20 M, 4 F, 36.1 ± 7.5/34.8 ± 6.9 yrs)	Marathon race	0 = no pain and 10 = worst possible pain	Pre/post	Reference: No *Reason for selection:* No	Bloating, nausea, urge to vomit, flatulence, urge to defecate, and stomach cramps	*Statistical method*: Mann–Whitney nonparametric U test for independent data analyzed as the sum of all moderate or worse symptoms (scale of 0–84; a maximum of 6 each day) and the total number of days in which any GIS ≥ 4 were reported *Data reported*: median and range
Pugh et al. [3]	11 trained runners (33.1 ± 10.4 yrs)	18 × 400-m interval efforts	10-point scale (0 = no symptoms and 10 = extreme symptoms	Pre/post	Reference: adapted from Pfeiffer et al. 2009 *Reason for selection:* No	Side stitch, Nausea, Bloating, urge to burp, Urge to vomit, Urge to defecate, Diarrhea, Flatulence, Stomach Cramps, Stomach upsets	*Statistical method*: Wilcoxon Signed Rank tests using means *Data reported*: median and range
Pugh et al. [110]	10 recreationally active males (24 ± 4 yrs)	60-min treadmill run at 70% $\dot{V}$ O_2max_ (30 °C)	10-point scale, ranging from 0 (“no problem at all”) to 9 (“the worst it has ever been”)	Post/24-h post	Reference: adapted from Pfeiffer et al. 2009 *Reason for selection:* No	Side stitch, bloating, urge to defecate, diarrhea, flatulence, stomach cramps, stomach upsets, intestinal cramps, urge to burp, nausea, urge to vomit, dizziness, shivering, heart burn	*Statistical method*: Magnitude-based inferences *Data reported*: median and range
Pugh et al. [111]	7 trained cyclists (23 ± 4 yrs)	2 h endurance cycling	0 -9 = no symptoms, and 9 = very severe symptoms, > 4 regarded as moderate	30-min	Reference: No *Reason for selection:* No	Bloating, nausea, urge to vomit, and urge to defecate	*Statistical method*: Students paired t-test *Data reported*: mean ± SD
Peters et al. [112]	155 well-trained males and females (30–49 yrs)	4 consecutive days of walking (203-km for males, 164-km for females)	Prevalence was categorized in “never,” “sometimes,” “often,” and “always” (“sometimes” was defined as “,50% of the trainings/competitions,” “often,” as “.50% of the trainings/competitions”	During	Reference: No *Reason for selection:* No	Nausea, belching, heartburn, chest pain, and vomiting) and of the lower part (GI cramps, bloating, diarrhea, flatulence, urge to defecate, and defecation)	*Statistical method*: Mann-Whitney test *Data reported*: mean ± SD
Pfeiffer et al. [4]	221 endurance athletes	2 Ironman triathlons, half-Ironman, a marathon, a100/150-km cycle race	10-point scale, 0 “no problem at all” to 9 “the worst it has ever been.” GIS that were scored were classified as “serious”	Post	adapted from Pfeiffer et al. [8]	Reflux/heartburn, belching, bloating, stomach cramps/pain, nausea, vomiting, intestinal/lower abdominal cramps, flatulence, urge to defecate, side ache/stitch, loose stool, diarrhea, intestinal bleeding, dizziness, headache, muscle cramp, urge to urinate)	*Statistical method*: Wilcoxon's signed-rank tests *Data reported*: Mean ± SD, minimum and maximum scores
Pettersson et al. [113]	12 elite cross-country ski athletes (6 F; 24.8 ± 5.3 6 M; 25.6 ± 4.7 yrs)	120-min of submaximal roller-skiing (69.3 ± 2.9% of $\dot{V}$ O_2peak_) in −5 °C. The maximal TT (2000 m for females, 2400 m for males) immediately followed the 120-min submaximal bout	rated on a 0–20 scale (0 = no symptoms, 10 = neutral, 20 = worst conceivable symptoms) and a level of digestive comfort was provided (0 = extremely uncomfortable, 10 = neutral, 20 = extremely comfortable)	20-min	Reference: Le Nevé et al. [114] *Reason for selection:* No	Gas, nausea, stomach rumbling, urgency to have a bowel movement and abdominal pain	*Statistical method*: Two-way ANOVA *Data reported*: mean ± SD
Ragone et al. [115]	10 male jiu-jitsu athletes (22.2 ± 3.9 yrs)	MVC and ISO test	The NRS (scale 0–10, with 0 reflecting no gastrointestinal discomfort and 10 indicating the most severe gastrointestinal discomfort)	20-min	Reference: Jeukendrup et al. [9], Dworkin et al. [116] *Reason for selection:* No	Nausea, stomach cramps, flatulence, belching, bloating and diarrhea	*Statistical method*: Friedman's *Data reported*: mean ± SD
Rauch et al. [117]	28 endurance runners (36 (32–43) yrs)	3 h steady-state running protocol	mVAS, 10-point rating scale, each point indicative of 10 mm	Pre/during/post	Reference: Gaskell et al. [19] *Reason for selection:* No	Belching, heartburn, stomach Bloating, upper abdominal pain, urge to regurgitate, and/or Actual regurgitation, flatulence, Lower abdominal bloating, lower abdominal pain, urge to Defecate, and abnormal defecation (e.g. loose watery stools, diarrhea, and/or fecal blood loss)	*Statistical method*: Mann–Whitney U test *Data reported*: Summative accumulation, mean ± SEM
Rauch et al. [118]	16 endurance athletes (9 M, 7 F; 37 ± 9 yrs)	3 h running at 60% $\dot{V}$ O_2max_ in 30 °C	mVAS, 10-point rating scale	Pre/during/post	Reference: Gaskell et al. [19] *Reason for selection:* No	Overall gut discomfort, belching, heartburn, stomach bloating, stomach pain, urge to regurgitate, regurgitation, projectile vomiting, flatulence, lower abdominal bloating, urge to defecate, left and right intestinal pain, and defecation with or without abnormalities, nausea, dizziness, and stitch	*Statistical method*: Wilcoxon signed ranks *Data reported*: Incidence and summative accumulation
Ramos-Campo et al. [119]	28 cyclists (27 ± 4 yrs)	Road cycling simulation - 8-min TT performance + 30-s TT + 4.5 hr of outdoor cycling + a second 8-min TT + a 30-s TT	Gut discomfort was evaluated using a VAS (from 0 mm [none discomfort] to 100 mm [maximum discomfort])	Pre/during/post	Reference: No *Reason for selection:* No	Did not state	*Statistical method*: Repeated measures ANOVA *Data reported*: mean ± SD
Rowe et al. [120]	11 trained males (29 ± 6 yrs)	120-min SS runs at 68% $\dot{V}$ O_2max_, followed by a 5-km TT.	10 point scale ranging from 1 (no problem at all) to 10 (the worst it has ever been), and a score of ≥ 5 was classified as having severe GIS	30-min	Reference: Jeukendrup et al. [9] *Reason for selection:* No	Upper, lower & systemic GI symptoms	*Statistical method*: Wilcoxon matched-pairs signed rank test *Data reported*: mean ± SD
Reynolds et al. [121]	11 trained runners (9 M, 2 F; 32 ± 8 yrs)	13.1 mile run on an outdoor running course	0 = no symptom,10 = max symptom, scores ≥ 5 were classed as severe	Pre/during/post	Reference: Jeukendrup et al. [9] *Reason for selection:* No	Hunger, thirst, nausea, stomach fullness, stomach bloatedness, stomach cramps, abdominal Stitch, flatulence, urge to belch, urge to vomit, urge to urinate and urge to defecate	*Statistical method*: Wilcoxon signed rank test *Data reported*: median (IQR)
Reynolds et al. [122]	9 male trained cyclists ( 24 ± 7 yrs)	2 h at ~55% Wmax (preload), followed by a TT designed to last ~15 min	0 = no symptom,10 = maximum symptom, scores ≥ 5 were classed as severe)	Pre/during/post	Reference: Jeukendrup et al. [9] *Reason for selection:* No	Hunger, thirst, nausea, stomach fullness, stomach bloatedness, stomach cramps, abdominal Stitch, flatulence, urge to belch, urge to vomit and urge to defecate	*Statistical method*: Wilcoxon signed rank test *Data reported*: median (IQR)
Roca Rubio et al. [123]	41 endurance trained (11 F, 30 M, 29.1 ± 6.2 yrs)	60-min treadmill run at 80% $\dot{V}$ O_2max_	0 to 7 Likert scale (0—no symptoms; 1—very mild; 2–4—mild; 4–6—moderate; 7—severe)	Post exercise	Reference: No *Reason for selection:* No	Belching, Heart burn, Bloating, Chest pain, Nausea, Vomit, Abdominal pain, Flatulence, Urge to defaecate, Diarrhea, Stitches, Bloody diarrhea	*Statistical method*: sCCA *Data reported*: Severity (reported by *n* (%))
Rowlands et al. [124]	10 well-trained mountain-bike riders (7 M, 3 F; 33 ± 9 yrs)	94-min high-intensity intervals followed by 10 maximal sprints	13.5 cm vertical line. 0, nothing at all; 0.5, extremely weak;1, very weak; 2, weak or mild; 3, moderate; 5, strong; 7, very strong; 10, extremely strong; and 13.5, absolute maximum	During the race	Reference: Borg CR10 scale [125] *Reason for selection:* No	Abdominal cramps, nausea, diarrhea	*Statistical method*: standardized magnitude-based inferences *Data reported*: Mean effect ± 90% CL
Sareban et al. [126]	9 well-trained triathletes (38.8 ± 10.1 yrs)	60-min swim, 180-min bike exercise, and a 60-min all-out run	Participants completed a questionnaire asking for common GIS	pre bike, every 60-mins during the bike, and pre/post run	Reference: No *Reason for selection:* No	Bloating, flatulencies, urge for defecation, defecation, stomach cramps, pain, diarrhea	*Statistical method*: McNemar's exact test *Data reported*: number of participants with corresponding GIS
Scrivin et al. [127]	11 endurance trained participants (6 M, 5 F, ≥ 18 yrs)	3 h steady-state running protocol	mVAS, 0 = no symptoms, 1–4 mild GIS, 5–9 severe GIS and 10 extremely severe GIS	Pre, during (15 mins) and post	Reference: Gaskell et al. [19] *Reason for selection:* No	Belching, heartburn, bloating, stomach pain, urge to regurgitate, regurgitation, projectile vomiting, flatulence, bloating, urge to defecate, intestinal pain, abnormal ­defecation, nausea, dizziness, abdominal stitch	*Statistical method*: Wilcoxen signed ranks test *Data reported*: Summative accumulation and participant range
Schreiber et al. [128]	27 male cyclists (28.3 ± 5.6 yrs)	$\dot{V}$ O_2max_ test and TTF test at 85% of maximal power	The online questionnaire which was specifically chosen for its unique relevance to endurance athletes.	Pre/during/post	Reference: Peters et al. [112] *Reason for selection:* No	Nausea, belching, heartburn, chest pain, vomiting, cramps, bloating, diarrhea, flatulence, urge to defecate and defecation	*Statistical method*: Mann-Whitney test *Data reported*: mean ± SD
Schroer et al. [129]	8 cyclists (22.3 ± 5.6 yrs)	120-min of constant-load cycling (55% of PP) followed by a 30-km TT	10-point scale (1 = not at all, 10 = very, very much)	30- and 120 mins of constant-load cycling and at 20- and 30-km of the TT	Reference: Jentjens et al. [23] *Reason for selection:* No	Stomach problems, GI cramping, bloated feeling, diarrhea, nausea, dizziness, headache, belching, vomiting, and urge to urinate or defecate	*Statistical method*: ANOVA *Data reported*: frequency table for severe symptoms (a score of ≥ 5)
Scott et al. [130]	13 males (21 ± 2 yrs)	2000-m rowing performance	1–10 VAS		Reference: No *Reason for selection:* No	Did not state	*Statistical method*: two-way repeated-measures ANOVA *Data reported*: mean ± SD
Seepika et al. [131]	8 male half marathon runners (33.25 ± 3.19 yrs)	Running TTE	VAS for GIS	Pre/mid/post	Reference: No *Reason for selection:* No	Did not state	*Statistical method*: two-way ANOVA *Data reported*: mean ± SD
Shannon et al. [132]	14 trained male cyclists (43 ± 15 yrs)	40 km TT	9-item, 10-point Likert Scale, (0 = no symptom, and 10 = severe symptom)	Pre/post	Reference: Carr et al. [43] *Reason for selection:* No	Nausea, flatulence, stomach cramping, belching, Stomach-ache, bowel urgency, diarrhea, vomiting, and Stomach bloating	*Statistical method*: Wilcoxon signed rank test *Data reported*: peak severity score (arbitrary units on 0–10 scale)
Snipe et al. [133]	24 endurance runners (13 M, 11 F; 35 ± 7 yrs)	2 h run at 60% $\dot{V}$ O_2max_ in 35 °C	A 10-point Likert-type rating scale was used to quantify self-perceived GIS, with 0 indicative of no symptoms to 10 indicative of extreme symptoms warranting activity withdrawal	10-min	Reference: Costa et al. [47] *Reason for selection:* No	Belching, heartburn, bloating Upper abdominal pain, urge to regurgitate, regurgitation, flatulence, urge to defecate, abdominal pains, abnormal defecation, nausea, dizziness, abdominal stitch	*Statistical method*: Friedman's test *Data reported*: Summative accumulation and range
Snipe et al. [134]	11 endurance runners (6 M, 5 F; 31 ± 6 yrs)	2 h run at 60% $\dot{V}$ O_2max_	Modified visual analog scale (incidence of severe symptoms (≥5 on the 0–10 modified visual analog scale)	Mid/post	Reference: Bengtsson et al. [17] *Reason for selection:* No	Belching, heartburn, bloating, stomach pain, urge to regurgitate, regurgitation, projectile vomiting, flatulence, bloating, urge to defecate, intestinal pain, abnormal ­defecation, nausea, dizziness, abdominal stitch	*Statistical method*: Friedman's test *Data reported*: accumulative score & individual participant range
Stuempfle & Hoffman [2]	272 runners	161-km Western States Endurance Run	0–4 scale (none = 0, mild = 1, moderate = 2, severe = 3, very severe = 4)	Post	Reference: No *Reason for selection:* No	Reflux/heartburn, belching, stomach bloating, stomach cramps/pain, nausea, vomiting), lower (intestinal cramps/pain, flatulence, side ache/stitch, urge to defecate, loose stool/diarrhea, intestinal bleeding/bloody feces)	*Statistical method:* GIS frequency across segments for finishers was done with Cochran's Q *Data reported*: Frequency & severity of GIS
Specht et al. [135]	11 physically active individuals (18-45 yrs)	1 h run (35 °C, 20%–60% RH) at 60% $\dot{V}$ O_2peak_	mVAS scale (incidence of severe symptoms (≥5 on the 0–10)	Pre/post	Reference: Gaskell et al. [19] *Reason for selection:* No	belching, heartburn, upper abdominal bloating, stomach pain, urge to regurgitate, flatulence, lower abdominal bloating, urge to defecate, abnormal stool, nausea, dizziness, stitch	*Statistical method*: Wilcoxon signed-rank test *Data reported*: Incidence (%) and median (range)
Sheahen et al. [136]	12 active males (22 ± 3 yrs)	Cycling (45-min, 70% $\dot{V}$ O_2max_)	0-4 Likert scale. No definitions provided	5-min	Reference: Hall, [24] *Reason for selection:* No	Belching, nausea, side stitch, abdominal cramps, urge to defecate	*Statistical method*: Friedman test and Wilcoxon matched-pairs signed rank test *Data reported*: median & 5th to 95^th^ percentile
Shing et al. [137]	10 trained male runners (27 ± 2 yrs)	TTE run, 80% VT	1–4 scale (1 = no/hardly any complaint, 4 = very severe complaint)	10-mins	Reference: No *Reason for selection:* No	Belching, nausea, side stitch, abdominal cramps and urge to defecate	*Statistical method*: paired t-tests *Data reported*: mean ± SE
Smith et al. [138]	37 female cyclists/triathletes (35 ± 6 yrs)	Virtual cycling race [19.5 km TT per week across a one-month period (totaling four races).	1 = no symptoms, 10 = extreme symptoms	Pre/post	Reference: Pugh et al. [109] *Reason for selection:* No	Nausea, Urge to vomit, Vomiting, Belching, Bloating, stomach pains/cramps, gastric acidosis, constipation, diarrhea, urge to defecate, gas	*Statistical method*: Repeated measures correlations *Data reported*: % incidence and number of “moderate” symptoms
Stellingwerff et al. [139]	23 healthy male cyclists (29 ± 7 yrs)	2 h of SS cycling (50% $\dot{V}$ O_2peak_)	This questionnaire asked 16 graded questions on a 10-point scale from 0 (none) to 9 (worst experienced)	Post	Reference: Pfeiffer et al. [4]; Pfeiffer et al. [8] *Reason for selection:* No	Reflux/heartburn, severe belching, bloating, stomach pain/cramps, vomiting, and nausea	*Statistical method*: paired t-tests *Data reported*: mean ± SD
Stocks et al. [140]	13 participants (10 M, 3 F, 30 ± 7 yrs)	30-km classic technique roller-skiing TT on a treadmill	GI discomfort was assessed using a modified Borg scale, where scores of 6 and 20 represent no discomfort and maximum discomfort, respectively	5km	Reference: McGawley et al. [141] *Reason for selection:* No	Not stated	*Statistical method*: two-way repeated measures ANOVA *Data reported*: Mean
Stubbs et al. [142]	42 participants (25.9 ± 5.0 yrs)	195 min (3.25 h) of continuous cycling, 3x 65-min blocks, with both low intensity (40-min at 40% W_max_) and high intensity (25 min alternating between 80% and 60% W_max_ every 5 min)	11 symptoms were given a score between 0 (not present) and 8 (unbearable)	Pre/post each drink	Reference: Pfeiffer et al., [4] *Reason for selection:* No	Reflux/heartburn, severe belching, bloating, stomach pain/cramps, vomiting, and nausea	*Statistical method*: McNemar's test was used to compare incidence (symptom vs. no symptom) between paired test conditions *Data reported*: Total symptoms reported
Sutehall et al. [143]	8 well-trained endurance athletes	four bouts of prolonged running (105 min)	0–9 scale (0 = none at all, 9 = the worst ever)	During exercise	Reference: Gaskell et al. [19] *Reason for selection:* “based on a previously validated questionnaire” (used a different scale)	Reflux or heartburn, belching, bloating, stomach pain or cramps, vomiting, and nausea, lower abdominal cramps, flatulence, urge to defecate, and diarrhea	*Statistical method*: Friedman and Wilcoxon signed-rank test *Data reported*: summative value per symptom and incidence (%)
Taylor et al. [144]	Moderately trained (16 M, 4 F, 29 ± 4 yrs)	70-min run at 70–90% of $\dot{V}$ O_2max_	mVAS scale (incidence of severe symptoms (≥5 on the 0–10)	10-mins	*Reference and reason for selection:* Validated as a reliable measure of GIS during exercise (Gaskell et al. [19]),	Belching, heartburn, bloating, stomach pain, urge to regurgitate, regurgitation, projectile vomiting, flatulence, bloating, urge to defecate, intestinal pain, abnormal ­defecation, nausea, dizziness, abdominal stitch	*Statistical method*: Friedman's non-parametric tests *Data reported*: Sum of each participant's aggregate scores and individual participant range
Too et al. [145]	11 male (29.3 ± 7.9 yrs)	80-min (75% $\dot{V}$ O_2max_) treadmill running followed by a 5 km TT	GIDQ created by our lab. The GIDQ included 7 categories, rated as 0 (none), 1 (mild), 2 (moderate), 3 (quite a lot), 4 (severe), 5 (very severe) and 6 (unbearable)	20-mins	Reference: No *Reason for selection:* No	Abdominal pain, nausea, heartburn regurgitation, belching, bloating, flatulence	*Statistical method*: two-way repeated measures ANOVA *Data reported*: mean ± SD
Umaña-Alvarado & Moncada-Jiménez [146]	11 males (30.2 ± 11.5 yrs)	10-km cross-country race	Did not state	Post	Reference: No *Reason for selection:* No	Abdominal pain/colic, abdominal swelling, reflux, urgency to defecate, abdominal/intestinal cramps, heartburn, nausea Vomit, appetite loss, flatulence, diarrhea	*Statistical method*: McNemar χ2 test *Data reported*: Frequency of GIS in participants
Ulluwishewa et al. [147]	16 individuals (8 M, 8 F; 18–50 yrs)	20-min run at 80% of maximal oxygen uptake on a 1% incline	Pre-run gut comfort was recorded on a scale of 1 (no discomfort) to 7 (very severe discomfort) Post exercise: modified version of the GIRS. Instead of rating the intensity, frequency and duration of the symptoms, they were simply asked whether they currently had any of the symptoms by answering “yes” or “no”	Pre/during/post exercise	Reference: Svedlund et al. [148] *Reason for selection:* No	Gut comfort, stomach-ache or pain, heartburn, acid reflux, hunger pains in stomach, nausea, burping, urgent bowel movement	*Statistical method*: Linear mixed model *Data reported*: mean (± SEM) gut discomfort scores
van Nieuwenhovern et al. [149]	98 well-trained (90 M, 8 F, 41 ± 8 yrs)	Competitive 18-km run	anchored 10-point scales (1 = not at all, 10 = very much)	During/post	Reference: Peters et al. [150] *Reason for selection:* No	Reflux, heartburn, bloating, upper abdominal cramp, vomiting, nausea, intestinal cramp, flatulence, urge to defecate	*Statistical method*: one-way ANOVA *Data reported*: Incidence is percentage of participants who reported presence of the complaint
van Venrooij et al. [20]	33 ultramarathon runners (46.1 ± 10.2 yrs)	60-km ultramarathon race	The severity of each complaint was reported on a 10 point scale from 1 (“no complaints”) to 10 (“never been worse”). Complaints with a severity < 5 were categorized as being mild, and > 4 as being serious	Pre/post	Reference: Jeukendrup et al. [9], Pfeiffer et al. 2012 *Reason for selection:* No	Belching, heartburn, bloating, vomiting, stomachache, flatulence, urge to defecate, stomach cramps, intestinal cramps and diarrhea	*Statistical method*: Kruskal-Wallis test *Data reported*: prevalence of GIS (%)
Whitham et al. [151]	7 recreationally active males (28.5 ± 5.5 yrs)	Preloaded (15 min at 65% $\dot{V}$ O_2max_) TT of 45-min	GIS and thirst using a VAS	Pre/post	Reference: Clarkson et al. [152]; Peters et al. [150] *Reason for selection:* No	Did not state	*Statistical method*: two-factor, repeated-measures ANOVA No data reported
Willems et al. [153]	12 recreationally active males (28 ± 6 yrs)	1 h run at 65% $\dot{V}$ O_2max_ (34.1 ± 0.1 °C, 40.8 ± 0.2% RH)	An adapted VAS 0-10 (0 = very mild symptoms, 5 = severe symptoms, 10 = extremely severe symptoms)	Pre/post	Reference: Gaskell et al. [19] *Reason for selection:* Adapted from Gaskell et al. [19]	Belching, heart burn, stomach bloating, stomach pain, urge to vomit, vomiting, flatulence, urge to defaecate, lower abdominal pain, nausea, dizziness, stitch	*Statistical method*: Mann–Whitney test *Data reported*: Summated scores
Wilson [154]	186 endurance athletes (84 M; 41 (33–50), 102 F; 38 (31–49) yrs)	In-house races	GIS were dichotomized into binary variables using a threshold of three or more on the 0–10 severity scale (0–2 = none, 3–10 = presence of GI distress)	Pre/post	Reference: No *Reason for selection:* No	Nausea, regurgitation/reflux, stomach fullness, abdominal cramps, gas/flatulence, urge to defecate	*Statistical method*: logistic regression models *Data reported*: odds ratios & prevalence (%)
Wilson [155]	150 runners (74 M; 46.5 ± 10.6, 76 F; 41.0 ± 11.7 yrs)	30-days of endurance training	0 to 10 scale with descriptors of “no discomfort”, “moderate discomfort “and “unbearable discomfort” anchored at 0, 5 and 10	Post	Reference: No *Reason for selection:* No	Nausea, regurgitation/reflux, stomach fullness, abdominal cramps, gas/flatulence, urge to defecate	*Statistical method*: Mann-Whitney U test & Spearman's rho correlations *Data reported*: correlations
Wilson [26]	53 triathletes	70.3-mile triathlon	Where 0 meant “no discomfort”, 5 meant “moderate discomfort” and 10 meant “unbearable discomfort”	Post	Reference: No *Reason for selection:* No	Nausea, regurgitation, stomach fullness, flatulence, lower abdominal cramps and urge to defecate	*Statistical method*: Spearman's rho coefficients *Data reported*: Prevalence of GI distress (%)
Wilson [156]	145 runners (70 M; 46.1 ± 10.7, F; 40.9 ± 11.8)	30-days of endurance training	Retrospective recall; 0 to 10 scale with descriptors of “no discomfort”, “moderate discomfort “and “unbearable discomfort” anchored at 0, 5 and 10	Post	Reference: No *Reason for selection:* No	Nausea, regurgitation/reflux, stomach fullness, abdominal cramps, gas/flatulence, urge to defecate	*Statistical method*: Mann-Whitney U test *Data reported*: recorded symptoms on % of runs
Wang et al. [157]	20 amateur marathon runners (16 M, 4 F, 25–50 yrs)	12-min Cooper running/walking distance test	GSRS, a 7-point scale ranging from 1 to 7	Pre/post	Reference: Asai et al. 2020, Tielemans et al. 2013, van Kerkhoven et al. 2008, Pugh et al. 2018 *Reason for selection:* This questionnaire was shown to be valid and reliable and has been used in large-scale surveys	Did not state	*Statistical method*: ANOVA *Data reported*: Prevalence of Aggregated score, mean and SD
Walter et al. [158]	6 healthy males (25 ± 5 yrs)	Running trial under hot conditions	GPSS; between 0 (no discomfort) and 10 (unbearable discomfort)	Pre/post	Reference: Wilson et al. [156] *Reason for selection:* No	Nausea, reflux/regurgitation, bloating, and stomach Fullness, abdominal cramps, gas/flatulence, and urge to defecate	*Statistical method*: Wilcoxon signed ranks *Data reported*: median ± IQR

Data presented as mean ± SD or mean (range), SS = steady state, VO2max = maximal oxygen uptake, VAS = visual analog scale, mVAS = modified visual analog scale, TTE = time to exhaustion, HRR = heart rate reserve, TT = time trial, RST = repeated sprint test, GSRS = Gastrointestinal Symptom Rating Scale, F = female, M = male, LT = lactate threshold, WAnT = Wingate Anaerobic Test, numerical classification scale = NRS, RE, Australian rules football = ARF, maximal voluntary contraction = MVC, isometric = ISO, time to fatigue = TTF, peak power = PP, gastrointestinal distress questionnaire = GIDQ. Standard error of the mean = SEM, standard deviation = SD, Analysis of Variance = ANOVA, confidence interval = CI, sparse canonical correlation analysis = sCCA.

## 3. Results

### 3.1. Study characteristics

One hundred and twenty-six studies were included, with a total of 3540 participants, ranging from *n* = 6 to *n* = 272, across a diverse range of exercise modes. Among these, 68 studies were running and 39 were cycling. Other exercise types included triathlons (*n* = 4), duathlon (*n* = 1), team sports (*n* = 6), and seven other actives, such as resistance training (*n* = 1), martial arts (*n* = 2), roller skiing (*n* = 2), cross-fit (*n* = 2), and rowing (*n* = 1), and exercise was conducted in both a laboratory environment (*n* = 96) and in the field (*n* = 30). Of the 126 studies, GIS (*n* = 93) was the most measured construct, while other studies assessed GI discomfort (*n* = 22), GI distress (*n* = 8), gastroparesis severity (*n* = 1), stomach comfort (*n* = 1), and bloatedness (*n* = 1). Table 1 presents an overview of the study characteristics.

### 3.2. GI scale characteristics

Fifty different scales were identified with a range of descriptors to measure the magnitude of GIS during exercise. Of these, 10 different Likert scale lengths were identified, with the most frequent being the 11-point scale. However, within this category, 12 different variations were identified due to the descriptors used for GIS magnitude, with studies modifying terms without justification (Table 2). Likert-type (category rating) scales were most common, with the number of studies for each scale length as follows: 4-point (*n* = 7), 5-point (*n* = 7), 6-point (*n* = 3), 7-point (*n* = 5), 8-point (*n* = 1), 9-point (*n* = 1), 10-point (*n* = 18), 11-point (*n* = 31), 21-point scale (*n* = 1), and 6-20 adapted Borg scale (*n* = 2). In addition, four different VAS lengths were identified, with the 100-mm VAS the most frequently employed. The following VAS formats were reported: 20-cm (*n* = 1), 100-mm/10-cm (*n* = 33), 13.5-cm (*n* = 1), and 133-mm (*n* = 1). Thirteen of the studies did not provide any information on the length of scale applied in their study and without reference to any previous scales.

**Table 2.** Likert and VAS scales used to collect GIS during exercise.

Study	Scales included and terminologies
**4-point scales**	
Mazur-Kurach et al. 2022	0–3 scale (0 = none, 1 = mild, 2 = moderate, 3 = severe)
Goedecke et al. 2005	0–3 scale (0 = to no symptoms to 3 = to symptoms severe enough to interfere with exercise performance)
Peters et al. 1999	“never”, “sometimes,” “often,” and “always” (“sometimes” was defined as “50% of the trainings/competitions,” “often,” as “50% of the trainings/competitions”)
Hall et al. 2013, Shing et al. 2014, Lee et al. 2018	1–4 scale (1 = hardly any complaint, 2 = moderate complaint, 3 = severe complaint, 4 = very severe complaint)
Lambert et al. 2008	1–4 scale (1 = very comfortable to 4 = very uncomfortable)
**5-point scales**	
Sheahen et al. 2018	0-4 Likert scale
Bennett et al. 2011, Stuempfle et al. 2015	0–4 scale (none = 0, mild = 1, moderate = 2, severe = 3, very severe = 4)
Exetbarria et al. 2021	0–4 scale (0 = never to 4 = all the time)
Haakonssen et al. 2014	1–5 scale (1 = very comfortable; 2 = comfortable; 3 = average comfort; 4 = uncomfortable; 5 = very uncomfortable)
Kuru et al. (2024)	0–4 scale (no problem = 0, very severe problem = 4)
**6-point scales**	
Biondich et al. 2016	0–5 scale (0 = none to 5 = unbearable)
Opheim et al. 2012	0–5 scale (0 = none to 5 = severe)
Chantler et al. 2022	0–5 scale (0 = nothing, 1 = just noticeable, not painful, 2 = noticeable, 3 = quite noticeable, mildly painful or disruptive, 4 = painful, quite disruptive, 5 = painful, may need medication)
**7-point scale**	
Too et al. 2012	0–6 scale (0 = none, 1 = mild, 2 = moderate, 3 = quite a lot, 4 = severe, 5 = very severe, 6 = unbearable)
Barber et al. 2020, Wang et al. 2024	7-point scale *(no other information provided)*
Ma et al. 2022	7 point scale (1 = absolutely no, 7 = absolutely yes)
Ulluwishewa et al. 2024	7 point scale (1 = no discomfort, 7 = very severe symptoms)
**8-point scale**	
Roca Rubio et al. 2024	0–7 scale (0—no symptoms; 1—very mild; 2–4—mild; 4–6—moderate; 7—severe symptoms)
**9-point scale**	
Stubbs et al. 2019	0–8 scale (0 = not present, 8 = unbearable)
**10-point Scales**	
Pfeiffer et al. 2012, Stellingwerff et al. 2013, Pugh et al. 2017(b), Edwards et al. 2021, Naderi et al. 2021, Rowe et al. 2022, Hart et al. 2022, Hanson et al. 2014, Lennon et al. 2024, Clayton et al. 2024, Kelly et al. 2023, Aragón-Vargas et al. 2023	0–9 scale (0 = no problem at all, 9 = “the worst it has ever been”)
Pugh et al. 2020	0–9 scale (0 = no symptoms, 9 = very severe symptoms)
Van Nieuwenhoven et al. 2005	1–10 scale (1 = not at all, 10 = very much)
Jentjens et al. 2002, Schroer et al. 2014	1–10 scale (1 = not at all, 10 = very, very much)
Sutehall et al. 2022	0–9 scale ( 0 = none at all, 9 = the worst ever)
van Venrooij et al. 2022	1–10 scale (1 = no complaints, 10 = never been worse)
**11-point Scales**	
Pugh et al. 2019	0–10 scale (0 = no pain, 10 = worst possible pain)
Pugh et al. 2017(a), Costa et al. 2017, Miall et al. 2018, Snipe et al. 2017, 2018, Smith et al. 2024	0–10 scale (0 = no symptoms, 10 = extreme symptoms)
Kahle et al. 2013, Durkalec-Michalski et al. 2025	0–10 scale (0 = GI distress at all, 10 = the most severe GI distress imaginable)
Ramos-Campo et al. 2024, Reynolds et al. 2023	0–10 scale (0 = no symptom, 10 = maximum symptom)
Ragone et al. 2020, Durkalec-Michalski et al. 2023	0–10 scale (0 = no GI discomfort, 10 = the most severe GI discomfort)
Leach et al. 2023, Bordoli et al. 2024, Shannnon et al. 2025	0–10 scale (0 = no symptom, 10 = severe symptom)
Morrison et al. 2014	0–10 scale (0 = no symptoms, 10 = severely uncomfortable)
Walter et al. 2021	0–10 scale (0 = no discomfort, 10 = unbearable discomfort)
Wilson et al. 2016; Wilson et al. 2018, Ehlert et al. 2023, Jones et al. 2025, Geaney et al. 2024	0–10 scale (0 = no discomfort, 5 = moderate discomfort, 10 = unbearable discomfort)
Oosthuyse et al. 2015(a), Oosthuyse et al. 2015(b)	0–10 scale (0 = nothing, 1 = very weak, mild; 2 = weak; 3 = moderate, 5 = strong, 7 = very strong, 10 = extremely strong)
Jeukendrup et al. 2000, Jonvik et al. 2019, Reynolds et al. 2022	0–10 scale (>5 = severe symptoms)
Mooren et al. 2020; Jardine et al. 2025	0–10 scale (*no other information provided*)
**6–20 Scales**	
Stocks et al. 2016	6–20 scale (0 = no discomfort 20 = maximum discomfort, respectively)
Cunniffe et al. 2016	6–20 scale (6 = none, 20 = very, very strong)
**0–20 Scales**	
Pettersson et al. 2019	0–20 scale (0 = no symptoms, 10 = neutral, 20 = worst conceivable symptoms)
**VAS (10 cm, 20 cm or 100 mm)**	
Gough et al. 2022	20 cm VAS - horizontal line, “no symptom” to the left and “severe discomfort” to the right
Burdon et al. 2023	10 cm (100 mm) VAS *(no other information provided)*
Baur et al. 2016 (adapted from above)	100 mm Likert scale (nothing at all, extremely weak, very weak, weak or mild, moderate, strong, very strong, extremely strong, and absolute maximum)
Lambert et al. 2001, Cameron et al. 2010, Miller et al. 2016, Gurton et al. 2021, Dobbin et al. 2021, Gough et al. 2024	100 mm VAS (0 = none; 100 = severe)
Costa et al. 2016, Campagnolo et al. 2017, Dudar et al. 2020	100 mm VAS (0 mm = no symptoms, 100 mm = extreme symptoms)
Nomura et al. 2025	10 cm VAS (mild = 1–3, severe = 4–5, very severe = 7–10)
Willems et al. 2025 (adapted from below)	0–10 VAS adapted from mVAS (below) (0 = very mild symptoms, 5 = severe symptoms, 10 = extremely severe symptoms)
Costa et al. 2020, Flood et al. 2020, Gaskell et al. 2020, Gaskell et al. 2022 (a,b), King et al. 2022; Ogden et al. 2022, Costa et al. 2023, Gaskell et al. 2023(a,b), Taylor et al. 2022, Martinez et al. 2024, 2025, Convit et al. 2024, 2025, Rauch et al. 2022, McCarthy et al. 2023, Rauch et al. 2025, Specht et al. 2025	100 mm mVAS (1–4 = mild symptoms, 5–9 = severe symptoms, 10 = extremely severe symptoms)
**Other**	
Rowlands et al. 2012	13.5 cm vertical line with regularly spaced verbal anchors and no numerical values
Lambert et al. 2007	133 mm scale indicating their level of comfort

VAS, visual analog scale; mVAS, modified visual analog scale.

**Figure 1.**
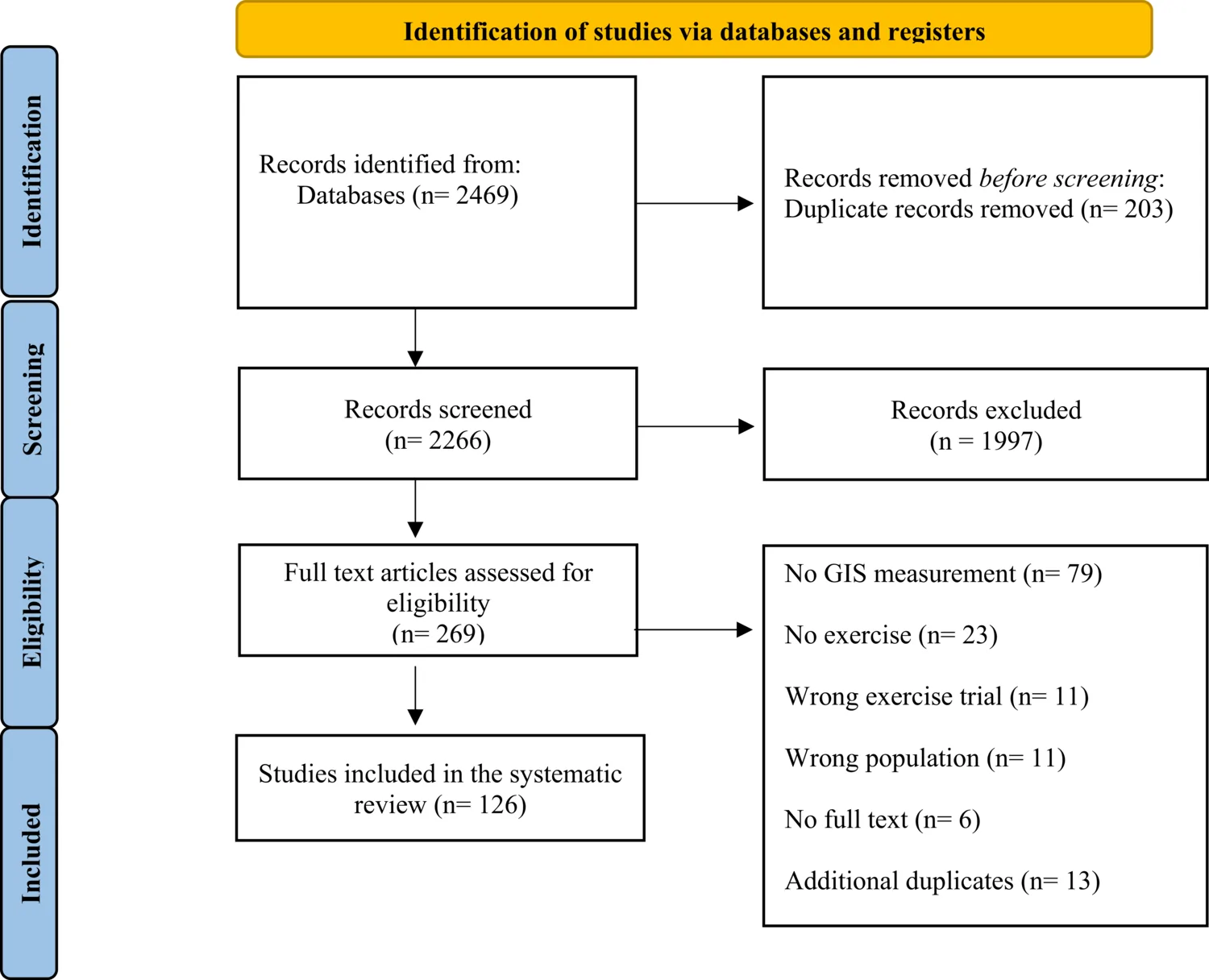
PRISMA flow diagram.

**Figure 2.**
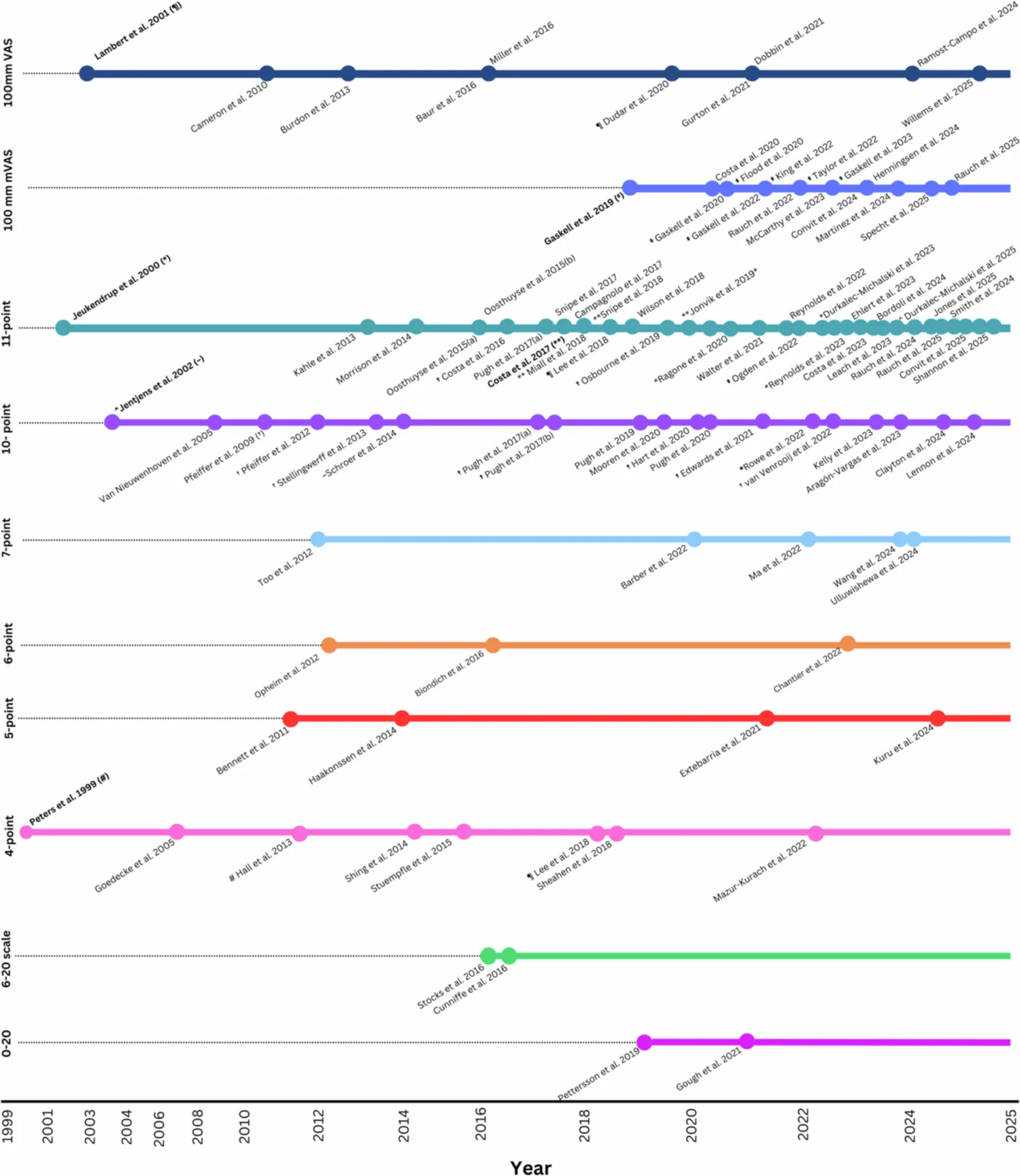
Timeline of use of Likert and VAS-type scales for measurement of gastrointestinal symptoms during exercise. Scales are categorized into eight sub-groups, based on scale length. Studies in bold text denote those which have been previously referenced. Of which the symbols represent reference to the following studies, ~[23], *[9], †[8], **[21], ‡[19], # [112], ¶[90]. Studies without a symbol did not justify their chosen scale with a citation from the literature or justified their chosen scale outside of the exercise literature; see Table 1 for details.

**Figure 3.**
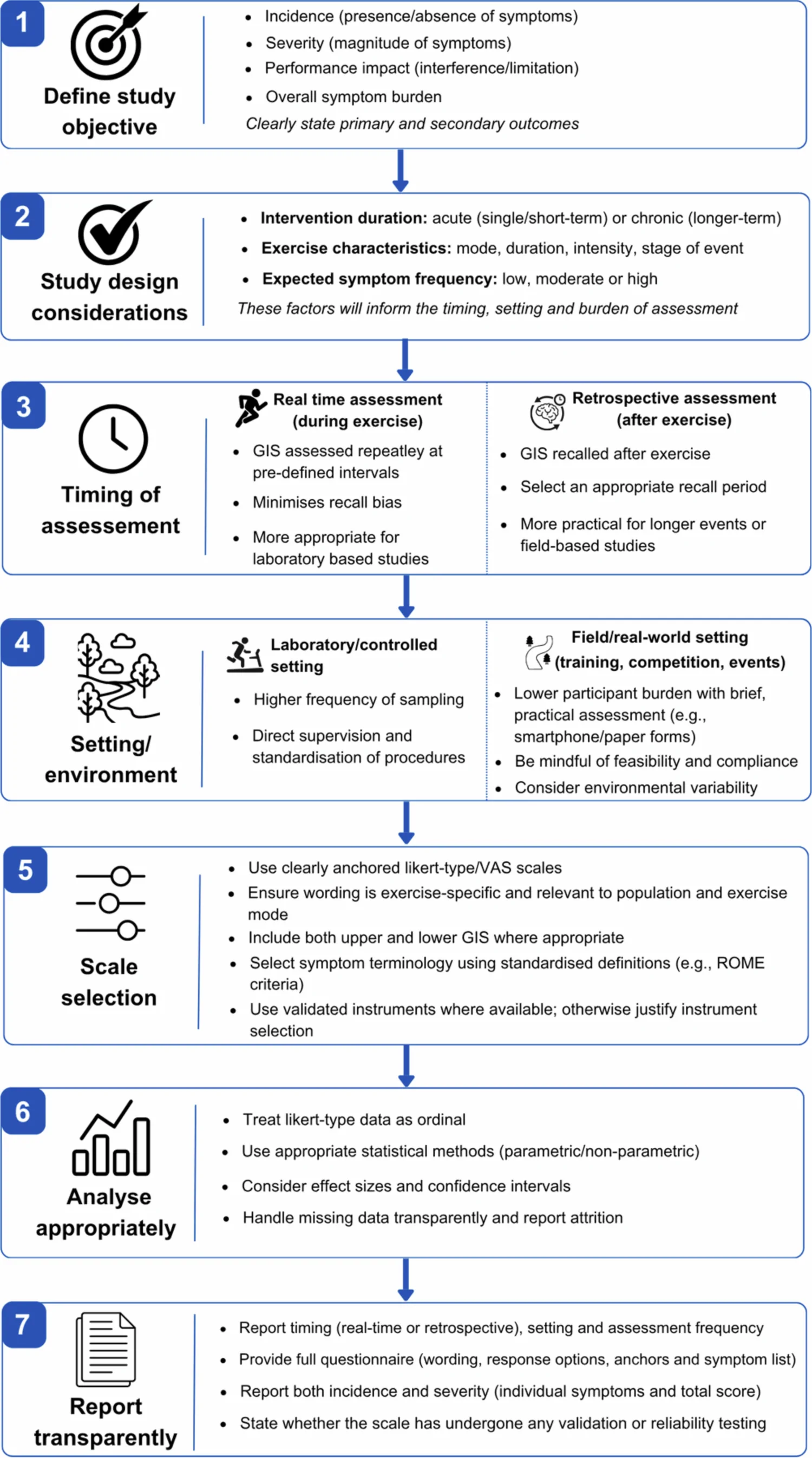
Proposed best-practice framework for the selection, implementation, analysis and reporting of gastrointestinal symptom (GIS) assessment methods in exercise research. VAS, visual analog scale.

The frequency of GIS recording varied among studies, ranging from pre- and post-exercise assessments to intervals spanning from 5 to 30 minutes. Laboratory-based studies (*n* = 95) predominantly utilized more frequent recording intervals compared to field studies (*n* = 30), such as triathlons or ultramarathons, where GIS assessments were commonly conducted pre- and post-exercise. Additionally, one study applied an open-ended approach, without specific questions at predefined time intervals [83]. The various exercise modes and environments revealed no discernible trend in scale selection (Table 1).

The number of symptoms recorded ranged from one to 14, with the most frequently recorded symptoms, in order of occurrence from most to least frequent, were descriptors of abdominal upset (pain, discomfort, ache; *n* = 109), bloating (*n* = 95), nausea (*n* = 92), the urge to defecate (*n* = 89), stomach cramps (*n* = 74), the urge to vomit (*n* = 71), belching/burping (*n* = 71), flatulence (*n* = 71), and heartburn (*n* = 53), stitch (*n* = 41), dizziness (*n* = 34), regurgitation (*n* = 28), urge to regurgitate (*n* = 24), intestinal cramp (*n* = 18), projectile vomiting (*n* = 18), fullness (stomach or gut; *n* = 15), intestinal pain (*n* = 12), bowel urgency (*n* = 7), abnormal defecation (*n* = 8), gastric bloating (*n* = 4), gastric acidosis (*n* = 4), abnormal stools (*n* = 2), loss of appetite (*n* = 2), Urge to burp (*n* = 2), gas (*n* = 2), stomach rumbling (*n* = 1), loss of desire to drink (*n* = 1), side-pain (*n* = 1), retrosternal discomfort (*n* = 1).

### 3.3. Historical analysis

The historical analysis of studies was presented to identify any trends in Likert-type scale lengths over time (Figure 2). There has been a substantial increase in GIS measurement through Likert-type scales and VAS, with 55% of research employing scales to measure exercise-associated GIS occurred since 2020. Despite this growth, no clear temporal trends were observed in the preference for scale type or length, indicating continued methodological heterogeneity throughout the study period. The mVAS was introduced in 2019 and demonstrated test–retest reliability; however, only 32% of studies published since 2021 referenced or applied this tool. Most studies did not explicitly justify their selection of Likert-type scales, often referencing prior sources that have been replicated or adapted from clinical scales in subsequent research (Figure 2).

### 3.4. Validation and reliability

Nine studies (10%) were cited by studies that had subsequently utilized or adapted their GI scale (Figure 2). Six studies referenced or adapted a previously validated gastroenterology scale, originally designed for non-exercise clinical settings. These included the Gastroparesis Cardinal Symptom Index [14], Visual Analog Scale for Irritable Bowel Syndrome [17], and GIS score for patients with functional dyspepsia [159]. Some studies referenced scales without appropriate full validation. For instance, one study [115] cited another [9] as a validated scale, despite no explicit validation stated in the original study. Additionally, other studies [68,85] referenced [19] as a “validated” scale. However [19], only conducted a test–retest reliability study, which was a modified VAS (mVAS) that had been fully validated for non-exercise contexts [17]. Importantly, only one study was identified that evaluated a retrospective questionnaire (0–10 Likert scale) specifically for GIS in an exercise context [156]. This scale assessed runners' symptoms based on recall over a 30-day period, rather than collecting data during or immediately after exercise bouts. No additional studies reporting formal evaluation of measurement properties for exercise-specific GIS scales were identified.

### 3.5. Statistical methods and reporting practices

To test the hypothesis of the study when using Likert scales (*n* = 86), 34% of the studies used ANOVA (*n* = 23) or paired t-tests (*n* = 6). Twenty-one percent of the studies opted for non-parametric alternatives, such as Friedman's test (*n* = 7) or the Wilcoxon signed-rank test (*n* = 11). Other statistical tests included the Mann–Whitney U-test (*n* = 7), McNemar's test (*n* = 3), Kruskal‒Wallis test (*n* = 3), magnitude-based inference (*n* = 2), Chi-square test (*n* = 2), Fisher's exact test (*n* = 1), Cochran's Q test (*n* = 1), Spearman's rho (*n* = 3), Pearson's product-moment correlation (*n* = 1), Spearman's rank test (*n* = 2), logistic regression (*n* = 2) and linear modeling (*n* = 3). Four studies did not state the statistical tools used and five did not report GIS results, despite detailing the scale in the methodology. For the VAS (*n* = 39), 26% of the studies used repeated measures ANOVA (*n* = 9) or paired t-tests (*n* = 1). In total, 38% of the studies opted for non-parametric alternatives such as Friedman's test (*n* = 6) or the Wilcoxon signed-rank test (*n* = 9). Other statistical tests included the Mann–Whitney U-test (*n* = 5), McNemar's test (*n* = 1), Kruskal–Wallis test (*n* = 2), and Magnitude Based Inferences (*n* = 1). Lastly, one study did not state the specific statistical tools used and a further three did not report the results of any GIS data, despite detailing the scale in the methodology.

The data reporting practices also varied across studies: for Likert scales, where parametric tests (ANOVA/t-test) were conducted, 56% of studies reported means (*n* = 16) along with SD (*n* = 12) or standard errors of the mean (SEM; *n* = 4) and 31% of studies reported accumulation scores (*n* = 9). For non-parametric analyzes (Friedman's or the Wilcoxon signed-rank test), 44% (*n* = 8) reported the median, while 33% reported accumulation scores (*n* = 6). Across Likert scales, data were also reported as confidence intervals (*n* = 1), frequencies (*n* = 5), correlations (*n* = 1), odds ratios (*n* = 1), and minimum/maximum scores (*n* = 1). For the VAS, where parametric tests (ANOVA/t-test) were conducted, 40% of studies reported means (*n* = 4) along with SD (*n* = 3) or SEM (*n* = 1). For non-parametric analyzes (Friedman's or the Wilcoxon signed-rank test), 23% of the studies reported median data (*n* = 2) with interquartile ranges (*n* = 1) or ranges (*n* = 1).

## 4. Discussion

To the authors' knowledge, this is the first study to systematically review and critically evaluate the historical use of Likert-type scales and VAS used to assess GIS during exercise and sport. The review specifically addresses the variations in scale taxonomy, scaling methods, symptom descriptors, statistical analysis, reporting, and validation of scales. By synthesizing the existing literature, this review aimed to highlight the key inconsistencies in GIS measurement and provide additional guidance on best practices.

### 4.1. Scale points

The progression of GIS scale selection in exercise settings across time (Figure 1) revealed key trends and inconsistencies in scale adoption. Notably, authors such as Pfeiffer [8] and Jeukendrup [9] were frequently cited in the literature, yet both studies provided limited justification for their chosen scales or references to prior research. Indeed, Jeukendrup [9] used an 11-point Likert scale to assess GIS during a long-distance triathlon, and seven subsequent studies referenced, applying unexplained variations of the 10-point and 11-point Likert scales. Across studies, no rationale was provided for the new scaling or terminology adopted. Another study [8] used a 10-point scale (0 = no problem at all, 9 = worst it has ever been) for assessing symptoms and was subsequently cited by 10 studies, either using the same scale or adapting it. This is noteworthy, as this scale considers any score >4 as “serious,” but does not provide full validation or reference to previous work to justify the scale's use. This pattern highlights a recurring theme in GIS measurement research, where there appears to be a lack of transparency and justification in scale selection and adaptation. Without clear reasoning to support the choice of the scale being used, the validity of the findings may be compromised, making it difficult to replicate studies, compare results, or draw meaningful conclusions. This overreliance on historical scales, often without adequate justification, emphasizes the need for more rigorous and transparent reporting practices to improve the quality and consistency of GIS research.

In an effort to assess GIS in exercise settings, Gaskell [19] adapted a 0–10 mVAS from the 100-mm VAS, originally validated for IBS in clinical settings [17]. This version of the scale was considered to be reliable for assessing GIS during endurance and, therefore, may be considered partially validated, representing progression in the methodological approaches used within this field. However, as the authors [19] identified, further research to provide a full validation study of the scale is necessary. It is also important for readers to interpret the 95% Limits of Agreement (95% LoA) data from this study, where total GIS randomly varied from ±25 (in the best case) to ±95 points (in the worst case). In practical terms, these limits of agreement suggest that studies aiming to detect relatively small changes in total GIS may struggle to distinguish true changes from measurement variability alone. For example, one study [57] reported relatively low total GIS scores (e.g. 2–4), whereas another study using the same scale [134] elicited substantially greater symptom burdens (e.g. 641–1370). Consequently, measurement error is likely to represent a larger proportion of the observed response in studies inducing only mild symptoms than in those producing more substantial GIS. These boundaries should be observed, and future researchers should consider adopting appropriate analytical goals before adopting new measurement tools [29], such as determining whether the scale can detect meaningful changes in GIS during exercise. Indeed, it is advisable that all potential users conduct their own reliability analysis as part of their study to facilitate this understanding, with attention to the scale of these non-parametric datasets, which has not yet been considered in the published literature. The modified GIS scale [19] scale has been cited by 32% of studies published since 2021 within the current review, with some referring to the scale as “validated” [18,68,160]. This raises a potential misunderstanding within the research community regarding the definition and requirements of a valid and reliable assessment tool. Consequently, the validity and reliability of many GIS scales currently used in exercise research remain uncertain.

### 4.2. Scale taxonomy

The categorization of symptom severity on numerical thresholds (e.g. 1–4 as “mild” and 5–9 as “severe”) is prevalent in exercise-based GIS research. Across the included studies, 25% classified symptoms as “mild,” 18% as “moderate,” and 40% as “severe,” although the numerical thresholds used to define these categories varied between studies. These descriptive classifications are widely used in clinical instruments to classify pain intensity [161,162] and standardizing symptom categorization settings [163–165]. While these classifications have been used as part of the Rome criteria [166] for diagnosing functional GI disorders (e.g. IBS), the gastrointestinal symptom rating scale (GSRS [167]) and its IBS-specific adaptation (GSRS-IBS [168]), the adoption of these terms to assess exercise-induced GIS lacks equivalent validation rigor. The GSRS-IBS established its severity thresholds through confirmatory factor analysis, demonstrating significant correlations between “severe” symptoms and rectal hypersensitivity [169] as well as quality-of-life impairments. While these descriptors can provide a threshold of symptom severity classification and should serve to anchor the participants' responses, their role extends beyond aiding participant responses. These classifications have also become markers used by researchers, clinicians, and practitioners to categorize symptoms, often assuming a universal understanding of their meaning. This raises two key considerations: first, participants may interpret these descriptors differently, leading to potential inconsistencies in symptom reporting; and second, that researchers may assume these terms have been understood and applied uniformly when analyzing responses. There is a need for greater scrutiny in how these descriptors are integrated into research methodologies, ensuring they are not only clear to participants but also appropriately contextualized for accurate data interpretation.

Despite their widespread use, the thresholds separating GIS severity classifications often lack rigorous statistical validation and appear to be more frequently based on convention than empirical evidence. Such validation could include (1) confirmatory factor analysis to verify unidimensionality, (2) convergent validity testing against gold-standard clinical or biomarker measures, or (3) sensitivity/specificity analysis to empirically anchor severity cut-points [28,170]. Unfortunately, these steps are often overlooked, which leads to inconsistency in the use and interpretation of GIS scales. Indeed, early attempts to quantify exercise-induced GIS, such as a frequency-based scale (“never,” “sometimes,” “often,” “always”), relied on retrospective athlete recall rather than direct measurement during exercise [112], potentially introducing recall bias [2]. Subsequent adaptations [24] applied a severity-based version (“hardly any” to “very severe complaint”), modified both descriptors and methodology by collecting real-time data during exercise. Notably, the transition from frequency-based to severity-based terminology reflects fundamentally distinct constructs, yet neither approach has been rigorously validated for athletic populations. Some authors [156], provided evidence of concurrent validation and reliability analysis of a retrospective GIS questionnaire; however, confirmatory factor analysis to verify questionnaire structure, convergent validity against independent clinical or biomarker measures, or sensitivity/specificity analyzes to empirically define severity thresholds were not included. The retrospective design means the questionnaire was not validated for capturing symptoms during exercise. Despite this, studies have employed this scale to collect GIS during exercise, even though it is not validated for this context [67,81]. Alarmingly, two studies have adapted the rating of perceived exertion [171] scale to assess GIS during exercise [58,140]. Although the 6–20 Borg scale was fully validated for ratings of perceived exertion (scaled from mean resting to maximum heart rate of 60 and 20, respectively), its effectiveness for GIS remains uncertain due to the lack of specific validation for this context. The rationale for adapting these scales to assess GIS during exercise is unclear, though it may stem from convenience and familiarity rather than empirical justification. Collectively, these scale adaptations, whether modifying response anchors, repurposing unrelated scales, or shifting constructs, prioritize convenience over validation. By treating scales as interchangeable templates rather than rigorously tested tools, researchers risk generating data that appear standardized but are in fact methodologically disjointed, ultimately undermining both scientific progress and clinical application for athletes.

The selection of scale terminology is a critical scientific issue, influencing the precision and consistency of data interpretation [11]. For example, the use of “5” as a threshold for categorizing “severe” symptoms on a 10- or 11-point scale is particularly prevalent, despite limited justification for this specific demarcation. A “severe” symptom is generally understood as one that significantly disrupts a person's ability to function normally, impacting their daily activities, physical performance, or overall well-being [1]. In the context of a 10- or 11-point scale, a score of 5 represents the midpoint between no GIS (0) and the most intense possible GIS (10 or 11). This midpoint is used as a practical cutoff because it suggests that the symptom is no longer mild or moderate but is of sufficient intensity to warrant attention or intervention. Jeukendrup [9] was one of the first studies to apply this 5-point threshold for classifying symptoms as severe but without detailed justification or reference to literature to support why a score of 5 was chosen. This lack of justification highlights a gap in understanding how this threshold was universally accepted and suggests the need for further examination of why a 5-point cutoff is regarded as appropriate and how it compares with other symptom assessment approaches. Future research should prioritize the empirical derivation of symptom severity thresholds rather than relying on convention. Potential approaches may include anchor-based methods, whereby symptom scores are linked to clinically or practically meaningful outcomes, such as reduced exercise capacity, interruption of training or competition, altered nutritional intake, or the requirement for medical attention. Importantly, the practical significance of GIS should extend beyond the numerical score itself. Thresholds should reflect the point at which symptoms meaningfully impair performance, compromise the ability to complete exercise, necessitate modification of nutritional strategies, or adversely affect athlete well-being. Defining the magnitude of symptoms is critical for understanding the impact of GIS on exercising individuals, as this helps researchers assess the degree to which symptoms are affecting health or performance and guide appropriate interventions. Furthermore, to ensure consistent understanding of GIS across the research and practitioner communities, it may be important to enhance the uniformity of descriptors used to define symptom magnitude. This requires that those administering the scales share a common understanding of how to explain these terms to participants and interpret the outcomes consistently.

### 4.3. Symptom descriptors

A wide variety of terminology and symptom variations are used across studies, which can complicate cross-study comparisons. Many studies fail to incorporate symptoms from clinically established gastroenterology literature, such as the ROME criteria. Gaskell and authors [19] recommended avoiding broad or imprecise terms like “gastrointestinal distress” or “burdened gut” to describe GIS, as these terms can lead to inconsistent interpretation. To improve clarity and consistency, 14 symptoms are recommended based on the ROME criteria. These symptoms are supported by evidence-based definitions [41,154,172–174]. The current variability in symptom terminology presents a significant challenge in GIS research. For instance, the term “stomach problems” [9,23,129] could lead to inconsistencies in symptom reporting and interpretation. Studies use a wide variety of terminology such as abdominal or stomach “pain” [98], “discomfort” [73], “ache” [91], or “cramp” [9]. These terms may yield different results depending on participants' perceptions and experiences, and their inconsistent application across studies complicates comparisons. Further, the overlap between symptoms, such as nausea and the urge to vomit, presents another challenge. Although these symptoms may be experienced as distinct by some individuals, they often evoke similar responses, leading to high correlations when included separately in scales. This overlap suggests that these symptoms, although treated as distinct, may represent the same underlying experience for many participants, potentially resulting in inconsistent or misleading data. Establishing clear and uniform definitions for symptoms is critical for facilitating accurate comparisons and enhancing the reliability of research findings. Future researchers may consider developing standardized symptom definitions informed by previous recommendations [18,19] and the ROME criteria [166] to ensuring consistency in symptom reporting and interpretation across studies.

### 4.4. Statistical methods and reporting

The treatment of Likert-type scale data in research remains a subject of debate, as these scales typically generate ordinal data, where intervals between points may not be equal [175]. Concerns about frequent data clustering, whether at extremes (e.g. ceiling effects) or central categories, challenge the use of parametric statistics and render the median a more robust measure of central tendency than the mean [176]. In contrast, VAS produce continuous data, which approximate interval measurement, potentially permitting more sensitive parametric analyzes [177]. However, both ordinal (Likert) and continuous (VAS) data are commonly treated with the same statistical analyzes across studies, despite their fundamental differences. As outlined in the results of the current review, both Likert and VAS-type scales were analyzed using various parametric and non-parametric methods, with no obvious pattern or consistent alignment between the choice of statistical method and the scale type. The lack of consensus on analysis type and reporting for both scales highlights methodological heterogeneity and an inconsistent recognition of data scale properties, which may increase difficulty for cross-study comparisons and reduce confidence in meaningful results. Additionally, when comparing the same scale (mVAS) across two studies with matched reporting (accumulative scoring), both a parametric (ANOVA) [94] and a non-parametric (Friedman test) [19] test were used for analysis. This difference also raises concerns about inconsistent assumptions, potential misinterpretation, and may limit comparability between results due to differing sensitivity and assumptions about data structure.

Parametric hypothesis testing, such as the use of ANOVA's and *t*-tests, is commonly applied when sufficient sample size is met [178–180]. In the current review, 85% of studies reported a sample size of ≥10 participants per group, and many studies use repeated-measures designs (typically pre-and post-intervention), where power would be enhanced by within-subject comparisons. However, it is common for GIS incidence reporting to be low, and studies will often report data of participants who were only symptomatic. This reduces the effective sample size and may increase the risk of type II error, thereby weakening the power and potentially biasing conclusions. To address these challenges, the use of alternative approaches may be required. For example, there was limited use of nested models across studies within this review (*n* = 3); however, cumulative link mixed models (CLMMs) have demonstrated accurate and robust analysis for ordinal data by properly modeling the ordered categories and accounting for hierarchical structures [181] and could therefore be considered by researchers. Although CLMMs are readily implemented in commonly used statistical software, researchers should consider the distribution of responses across symptom categories, as sparse data and floor or ceiling effects may influence model stability and interpretation [181].

For reporting GIS scale data, Gaskell and authors [19] have highlighted the need for consistent and comprehensive presentation of GIS metrics, providing detailed recommendations for researchers and practitioners. Specifically, the authors recommend reporting the incidence of GIS, derived from Likert-type or VAS, indicating the total percentage of participants who experienced GIS, regardless of severity or type [19]. It was also recommended that the frequency of severe GIS is treated separately, and individual symptoms are reported [18]. However, a critical issue in exercise-related GIS research is the lack of sensitivity in many scales, which often results in low numbers of reported symptoms or severity levels. Gaskell [19] proposed a method to enhance interpretability, suggesting the use of accumulated scores of incidence or severity. While these guidelines standardize GIS reporting (incidence, severity, symptom specificity), fundamental limitations persist in that existing scales (e.g. VAS, Likert) may either i) lack sensitivity to capture low-frequency, yet clinically significant symptoms or ii) continue to apply scale items (with equal weighting) that are not transferrable to exercise settings. Importantly, many of these scales are applied in research environments where exercise intensity and duration may be insufficient to provoke clinically relevant GIS; a minimum threshold of ~2 hours at 60% $\dot{V}$ O_2max_ in hot conditions or 3 hours in temperate settings is needed to induce GI disturbances with clinical and performance implications [182]. Indeed, low-frequency symptoms such as exercise-induced nausea or mild abdominal cramping, although often transient and sporadic, can still impair performance and have health implications. Such episodic symptoms may be under-detected by scales developed for chronic clinical populations, where symptom frequency and presentation can differ, and this limitation may also arise from the carryover of clinical scales without adaptation to exercise-specific symptoms. The proposed accumulated severity metric attempts to address this by aggregating transient events rather than dismissing them. However, this workaround does not resolve the core problem of scale validity. Indeed, this unweighted aggregation of scores alters the nature of the measurement by diluting its time resolution and ignoring the dispersion of scores about the mean. This approach creates a larger number to facilitate statistical analysis but has the potential to misinterpret the measurement. Moving forward, researchers could consider employing current reporting guidelines [18] while explicitly acknowledging these scale limitations. To avoid missing critical information, aggregated data, it would be useful to supplement this with individual-level responses to provide a more accurate representation of symptom variability.

## 5. Limitations

Several limitations of the present review should be acknowledged. First, only studies published in English were included, which may have resulted in the exclusion of relevant research reported in other languages. Second, the review was restricted to studies involving healthy adults aged ≥ 18 years; therefore, the findings may not be generalizable to pediatric populations or individuals with clinical GI conditions. Third, although multiple databases were searched, the use of four databases may have limited the identification of all relevant studies, particularly those indexed exclusively elsewhere. While reference list screening and citation tracking were undertaken to maximize study identification, symptom-specific terms (e.g. nausea, bloating, vomiting, and cramping) were not explicitly included within the search strategy. Consequently, studies indexed primarily using symptom-specific terminology rather than broader GI terms may have been underrepresented. Fourth, the review protocol was not prospectively registered, which may limit transparency regarding decisions made during the review process. Finally, although validation evidence was extracted and discussed where available, this review was not designed as a formal systematic review of measurement properties. Consequently, instrument-specific appraisal frameworks were not applied, and the synthesis of reliability and validity evidence was limited to the information reported within the included studies.

### 5.1. Future research recommendations

It is recommended that future research focus on adopting more standardized approaches to measuring exercise-induced GIS, incorporating the factors discussed herein. Researchers should carefully consider the context in which GIS is being assessed when selecting and implementing symptom assessment methods. Based on the findings of the present review, a best-practice framework is proposed to guide the selection, implementation, analysis and reporting of GIS assessment methods in future exercise research (Figure 3). Although this framework is not intended to replace formal instrument validation, it provides practical recommendations to improve methodological consistency and transparency across exercise studies and should be used alongside future validated exercise-specific instruments.

While GIS scales have been fully validated in clinical populations, they have not undergone equivalent validation for use during exercise. Future research should therefore prioritize the development and psychometric evaluation of an exercise-specific GIS instrument that captures the unique physiological, environmental and methodological characteristics of exercise. Such work should establish content validity, construct validity, reliability, responsiveness and interpretability across different exercise modalities and settings. In parallel, adoption of the proposed framework may improve consistency in the selection, implementation and reporting of GIS assessment, facilitating comparison between studies and supporting the accumulation of more robust evidence. Crucially, such a tool must be sensitive enough to detect the wide inter-individual variability in symptom presentation, ranging from athletes who report little to no symptoms to those with severe, performance-limiting GIS. Without this sensitivity, symptom burden may be underestimated or misclassified. A validated, standardized scale would improve comparability across studies and enhance the evaluation of interventions (e.g. dietary or hydration strategies). Further, researchers should select statistical methods that are appropriate for the properties of the data generated by a standardized tool. Likert-type scales produce ordinal data and may require non-parametric or ordinal modeling approaches (e.g. CLMMs for repeated measures), whereas VAS data may permit parametric analyzes when assumptions of interval measurement and normality are met [180,183]. To create a valid and reliable tool for measuring exercise-induced GIS, the following key principles should be considered for guiding development:

1. Validation of scale design and anchoring

- Begin with a clear validation framework tailored to the target population (e.g. athletes) and context (e.g. during or post-exercise), similar to previous established methodologies for assessing subjective responses in exercise settings [171,184]. Examples of structured scale development in related health contexts demonstrate the importance of a sequential approach incorporating content validity, factor analysis, and reliability testing prior to implementation [185].
- Assess test–retest reliability, construct validity, criterion validity, and responsiveness to change [28,29].
- Use *a priori* analytical goals to distinguish between real, meaningful changes and purely statistical ones [29].
- Ensure the scale is sufficiently sensitive to detect inter-individual variability without inflating false positives or masking true effects.
- Empirically derive symptom severity thresholds using clinically or practically meaningful outcomes, rather than relying on convention-based cut-offs.

2. Symptom terminology and clarity

- Use precise, standardized terms from gastroenterology literature (e.g. ROME criteria), that are clinically validated for GIS.
- Avoid ambiguous or lay terms; instead, ensure symptom labels align with clinical understanding and athlete experience.
- Consider whether symptoms are best rated on separate scales (e.g. nausea vs. urge to vomit) or collapsed into composite scores; this decision should be informed by the validation process outlined in the validation steps above.

3. Practicality and user experience

- Design the tool to be quick, unobtrusive, and feasible during or after exercise.
- Pilot test with the target population to refine usability and clarity.
- Balance sensitivity with low participant burden, especially in field settings.
- Consider the use of appropriate statistical models such as CLMM's that handle typical data complexities.

By addressing these considerations, future researchers can develop a standardized tool that not only facilitates meaningful cross-study comparisons but also improves the accuracy of GIS assessment in exercise settings. This could ultimately lead to more reliable insights and practical applications in both research and clinical practice. However, if a validated scale is not available, it may be advisable for researchers to carefully consider scale selection and explicitly justify their scale selection, whilst acknowledging the absence of validation as a limitation.

### 5.2. Conclusion

This current review provides a comprehensive evaluation of GIS measurement approaches used in exercise settings, highlighting both the historical development of symptom scales and the substantial heterogeneity that persists within the literature. Fifty different scales and 13 scale lengths were identified, with 10-point, 11-point, and 100-mm visual analog scales being the most frequently used. However, many studies relied on in-house tools or adaptations of published scales, often without explicit justification or evidence supporting their validity or reliability in exercise contexts. Variation in symptom terminology, scale descriptors, severity classifications, and statistical reporting further limits the comparability and interpretation of findings across studies. Until a fully validated exercise-specific GIS instrument becomes available, researchers should justify scale selection, adopt evidence-based symptom terminology, define severity thresholds transparently, and acknowledge the limitations associated with their chosen assessment approach. Although recent advancements have brought the field closer to standardization, the development and validation of an exercise-specific GIS measurement framework remains a key priority. Such a framework should incorporate clear symptom definitions, justified scaling methods, and rigorous psychometric evaluation to strengthen the consistency, interpretability, and translational value of GIS research in athlete health and performance settings.

## Data Availability

Available on request.
